# ADP-Hep-Induced Liquid Phase Condensation of TIFA-TRAF6 Activates ALPK1/TIFA-Dependent Innate Immune Responses

**DOI:** 10.34133/research.0315

**Published:** 2024-02-14

**Authors:** Liping Li, Jia Wang, Xincheng Zhong, Yaoyao Jiang, Gaofeng Pei, Xikang Yang, Kaixiang Zhang, Siqi Shen, Xue Jin, Gaoge Sun, Chaofei Su, Shuzhen Chen, Hang Yin

**Affiliations:** ^1^ State Key Laboratory of Membrane Biology, School of Pharmaceutical Sciences, Institute for Precision Medicine, Tsinghua-Peking Center for Life Sciences, Key Laboratory of Bioorganic Phosphorous chemistry and Chemical Biology (Ministry of Education), Tsinghua University, Beijing 100084, China.; ^2^ Department of Cancer Research, Institute of Medicinal Biotechnology, Chinese Academy of Medical Sciences and Peking Union Medical College, Beijing 100050, China.; ^3^ State Key Laboratory of Membrane Biology, Institute of Molecular Medicine, College of Future Technology, Peking University, Beijing, China.; ^4^ School of Life Sciences, Tsinghua University, Beijing, 100084, China.

## Abstract

The ALPK1 (alpha-kinase 1)-TIFA (TRAF-interacting protein with fork head-associated domain)-TRAF6 signaling pathway plays a pivotal role in regulating inflammatory processes, with TIFA and TRAF6 serving as key molecules in this cascade. Despite its significance, the functional mechanism of TIFA-TRAF6 remains incompletely understood. In this study, we unveil that TIFA undergoes liquid–liquid phase separation (LLPS) induced by ALPK1 in response to adenosine diphosphate (ADP)-β-D-manno-heptose (ADP-Hep) recognition. The phase separation of TIFA is primarily driven by ALPK1, the pT9-FHA domain, and the intrinsically disordered region segment. Simultaneously, TRAF6 exhibits phase separation during ADP-Hep-induced inflammation, a phenomenon observed consistently across various inflammatory signal pathways. Moreover, TRAF6 is recruited within the TIFA condensates, facilitating lysine (K) 63-linked polyubiquitin chain synthesis. The subsequent recruitment, enrichment, and activation of downstream effectors within these condensates contribute to robust inflammatory signal transduction. Utilizing a novel chemical probe (compound **22**), our analysis demonstrates that the activation of the ALPK1-TIFA-TRAF6 signaling pathway in response to small molecules necessitates the phase separation of TIFA. In summary, our findings reveal TIFA as a sensor for upstream signals, initiating the LLPS of itself and downstream proteins. This process results in the formation of membraneless condensates within the ALPK1-TIFA-TRAF6 pathway, suggesting potential applications in therapeutic biotechnology development.

## Introduction

Innate immunity stands as the primary defense mechanism against a spectrum of pathogens, encompassing fungi, bacteria, and viruses. Mammalian cells rely on a diverse array of germline-encoded pattern recognition receptors (PRRs) to detect pathogen-associated molecular patterns (PAMPs) [1–4], forming a crucial defense line. Recently, alpha-kinase 1 (ALPK1) and ADP-β-D-manno-heptose (ADP-Hep) have emerged as novel components of this intricate system, constituting a PRR and an effective PAMP in the ADP-Hep/ALPK1/TIFA axis, respectively [5]. ADP-Hep, a product of the lipopolysaccharide (LPS) biosynthetic pathway in gram-negative bacteria, directly binds to the N-terminal domain of ALPK1. This interaction induces the activation of the kinase domain of ALPK1, subsequently triggering TIFA phosphorylation. The phosphorylated TIFA, in turn, initiates the activation of TRAF6, enhancing its ubiquitin ligase activity [6]. Consequently, this cascade promotes the activation of the TAK1 and IκB kinase complex (IKK) through phosphorylation and ubiquitination events. The activation of these pathways culminates in the activation of the nuclear factor κB (NF-κB) pathway and the subsequent secretion of inflammatory cytokines.

TIFA, a crucial 21-kDa adaptor protein, assumes a central role as the primary host factor responsible for orchestrating inflammatory responses upon bacterial heptose recognition. Numerous reports have indicated that the overexpression of TIFA can elicit heightened transcriptional activity of NF-κB [6]. The human TIFA protein boasts distinctive features, including a threonine phosphorylation site (T9), a forkhead-associated (FHA) domain capable of specifically recognizing phosphoserine (pS) and phosphothreonine (pT) residues [7], and a C-terminal domain known to harbor a TRAF6 binding site. TIFA exhibits the ability to bind to both TRAF6 and TRAF2, thereby playing an important role in innate immunity [8,9]. Beyond its contributions to innate immunity, TIFA emerges as a pivotal mediator in the priming and activation of nucleotide-binding oligomerization domain-like receptor family pyrin domain-containing protein 3 (NLRP3) inflammasome [10]. Intriguingly, reports have highlighted TIFA’s involvement in supporting the progression of acute myeloid leukemia [11], further underscoring its multifaceted role in cellular processes.

During pathogen infection, the intriguing formation of TIFAsomes [12] has been documented through a TIFA-dependent cytosolic monitoring pathway. This intricate pathway is activated by ADP-Hep, a metabolite of LPS, thereby triggering the NF-κB signaling cascade [5]. Furthermore, TIFA oligomers have been identified in infections caused by *Shigella flexneri* and *Salmonella typhimurium* [12,13], underscoring their dependence on both TIFA and TRAF6. Notably, the formation of TIFAsomes is predominantly associated with infections by gram-negative bacteria, given their production of heptose [12]. These distinct speckles rely on phosphorylation at T9 of TIFA [14,15]. This phosphorylation event fosters “head-to-tail binding” interactions between different TIFA dimers, culminating in the formation of higher-order TIFA structures that efficiently drive downstream signaling events.

TRAF6, functioning as an E3 ligase, substantially amplifies the synthesis of K63-linked polyubiquitin chains through the ubiquitin E1 and E2 complex Ubc13/Uev1A. This process is imperative for activating TAK1 and IKK complexes [16–18]. Furthermore, in the enzymatic reaction, the supplementation of TRAF6 contributes to the generation of more extensive and prolonged K63-polyUb, as well as the formation of numerous and larger NEMO(NF-kappa-B essential modulator) liquid droplets [19]. Notably, TRAF6 assumes a pivotal role in the ALPK1/TIFA signaling pathway in response to ADP-Hep recognition [5]. This involvement hinges on TRAF6, with its binding motif situated at the C-terminus and encompassing Glu178 [20,21]. However, the binding site alone proves inadequate for recruiting a substantial quantity of TRAF6 to effectively activate downstream pathways. Consequently, the intricate mechanism elucidating how TRAF6 is recruited in large amounts by TIFA remains an area requiring further investigation.

In this study, a comprehensive investigation involving biophysical, biochemical, and cellular analyses conclusively affirms that ADP-Hep induces TIFA to establish a dynamic liquid–liquid phase separation (LLPS), as opposed to forming higher-order TIFA structures. The LLPS of TIFA is predominantly propelled by ALPK1, the pT9-FHA domain, and specific segments. Intriguingly, the E178A TIFA mutant exhibits LLPS, suggesting that binding to TRAF6 is not indispensable for the LLPS of TIFA. Simultaneously, we have substantiated that TRAF6 also undergoes phase separation during ADP-Hep-induced inflammation, and this phenomenon is universally observed across different inflammatory signal pathways. Notably, the membraneless condensates of TIFA-TRAF6 include essential components for ubiquitin chain synthesis, facilitating the efficient production of long ubiquitin chains. TAK1, TAB2, and NEMO are concentrated into the condensates, expediting their phosphorylation and ubiquitination processes and ultimately leading to robust activation of the inflammatory signaling cascade. Furthermore, our study employs a novel chemical probe (compound **22**) to validate the potential impact of TIFA LLPS in stimulating the ALPK1/TIFA/TRAF6 signaling pathway in response to small molecules. The findings reveal that the activation of this pathway is contingent upon the phase separation of TIFA.

Therefore, we present a superior organization of TIFA-TRAF6 and its downstream interacting proteins while recognizing PAMPs and DAMPs. This organization efficiently facilitates signal transduction and amplification.

## Results

### TIFA forms liquid-like condensates stimulated with ADP-heptose

**Fig. 1.**
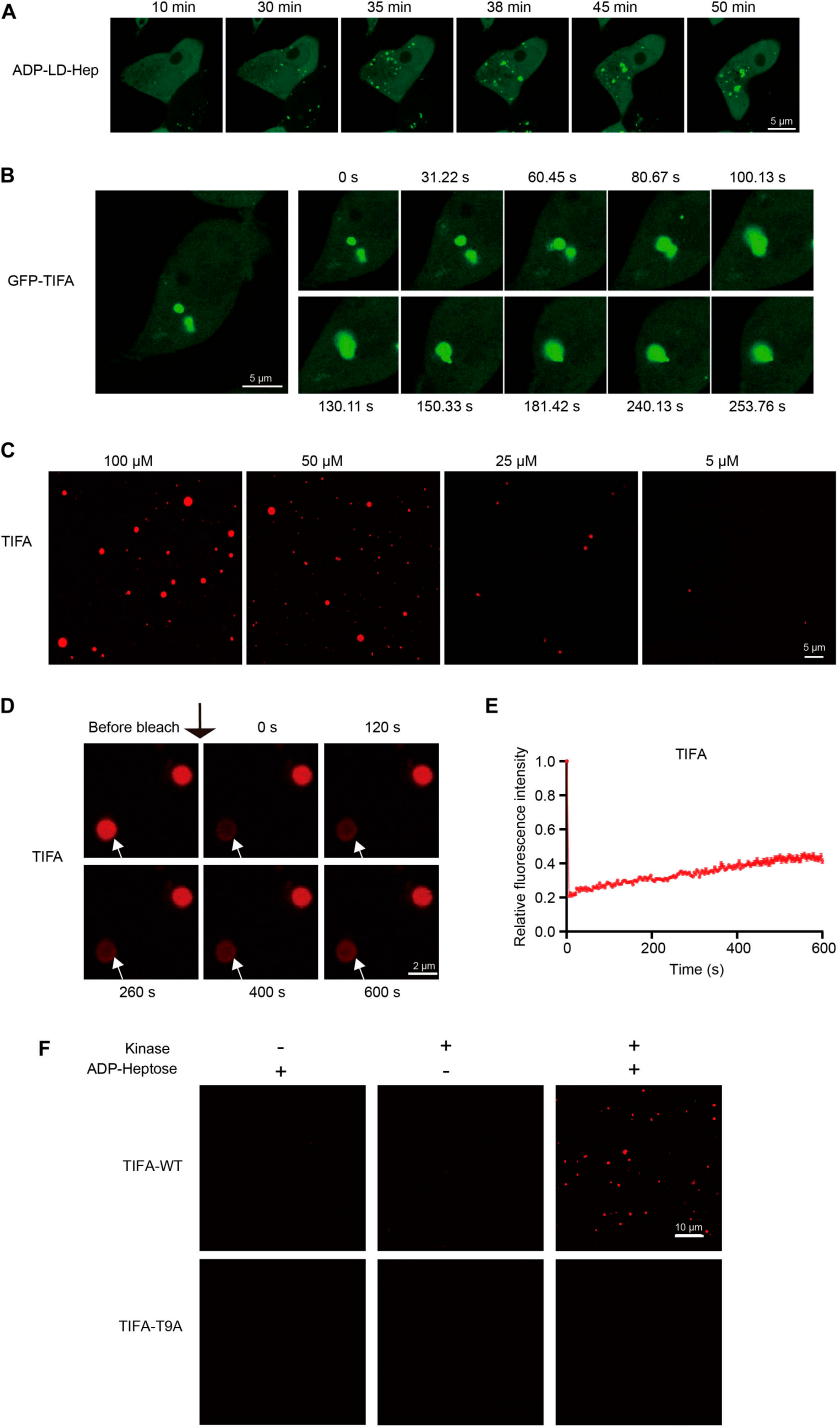
TIFA forms liquid-like condensates stimulated with ADP-heptose. (A) Representative live cell images of TIFA condensates in 293T cells stimulated with ADP-LD-Hep (10 μM) for 1 h. Scale bars, 5 μm. (B) Time-lapse micrographs of TIFA condensates formation and fusion (time 0 represents 60 min after ADP-LD-Hep treatment). Scale bars, 5 μm. The fusion events exist in all 3 fields examined. (C) In vitro phase separation assay of TIFA at various protein concentrations (5 to 100 μM). Scale bars, 5 μm. (D) FRAP of TIFA condensates. Time 0 represented the time of starting photobleaching pulse. Scale bars, 2 μm, *n* = 6 independent experiments. (E) FRAP of TIFA puncta. Data are means ± SEM (*n* = 4). (F) Representative images of TIFA-WT and TIFA-T9A condensates induced by ADP-LD-Hep. Scale bars, 10 μm.

TIFA has been observed to form condensates in cells stimulated with ADP-heptose or certain gram-negative bacteria [5,12,13]. However, the mechanism and function of such TIFA puncta are poorly understood. To investigate whether TIFA specks exhibit liquid-like characteristics, we utilized human embryonic kidney 293T cells (HEK 293T cells) due to their low TIFA expression. Initially, we overexpressed green fluorescent protein (GFP)-tagged TIFA (GFP-TIFA) in HEK 293T cells. Upon stimulation with ADP-LD-Hep (structures shown in Fig. S1A) for 30 min, the formation of TIFA specks (green) with 6 to 7 puncta per cell was observed, and dynamic changes in both number and size were detected through live-cell imaging tracking (Fig. 1A). Moreover, the percentage of cells containing puncta increased over a time-course treatment of ADP-LD-Hep at 10 μM (Fig. S1B). Time-lapse microscopy revealed that small TIFA liquid droplets fused and relaxed into larger droplets upon intersection, as evidenced by increased fluorescence intensities and elongated diameters (Fig. 1B). These results suggest that TIFA exhibits dynamic liquid-like behavior in cells stimulated with ADP-LD-Hep.

In additional experiments utilizing purified full-length TIFA protein from *Escherichia coli* BL21 (DE3) (Fig. S1C to E), it was confirmed that TIFA readily forms liquid droplets in vitro within 2 min of dilution into an appropriate phase-separation buffer. Increasing the TIFA concentration resulted in the rapid enlargement of droplets, indicating that higher TIFA concentrations promote LLPS (Fig. 1C). Fluorescence recovery after photobleaching (FRAP) experiments demonstrated slow fluorescence recovery for TIFA (Fig. 1D and E), suggesting the formation of gel-like condensates with relatively low fluidity. Subsequently, incubating a low concentration of TIFA protein (6 μM) and ADP-LD-Hep-treated cell lysates overexpressing ALPK1 or not for about 30 min showed that TIFA could still be phosphorylated at the T9 site and form enlarged liquid droplets in the presence of ALPK1 in vitro (Fig. 1F and Fig. S1F), confirming TIFA’s ability to undergo LLPS. These findings demonstrate that TIFA can undergo phase separation, not only forming condensates at the cellular level but also in vitro.

### ALPK1, the pT9-FHA domain, and the IDR segment are necessary for TIFA liquid condensation

**Fig. 2.**
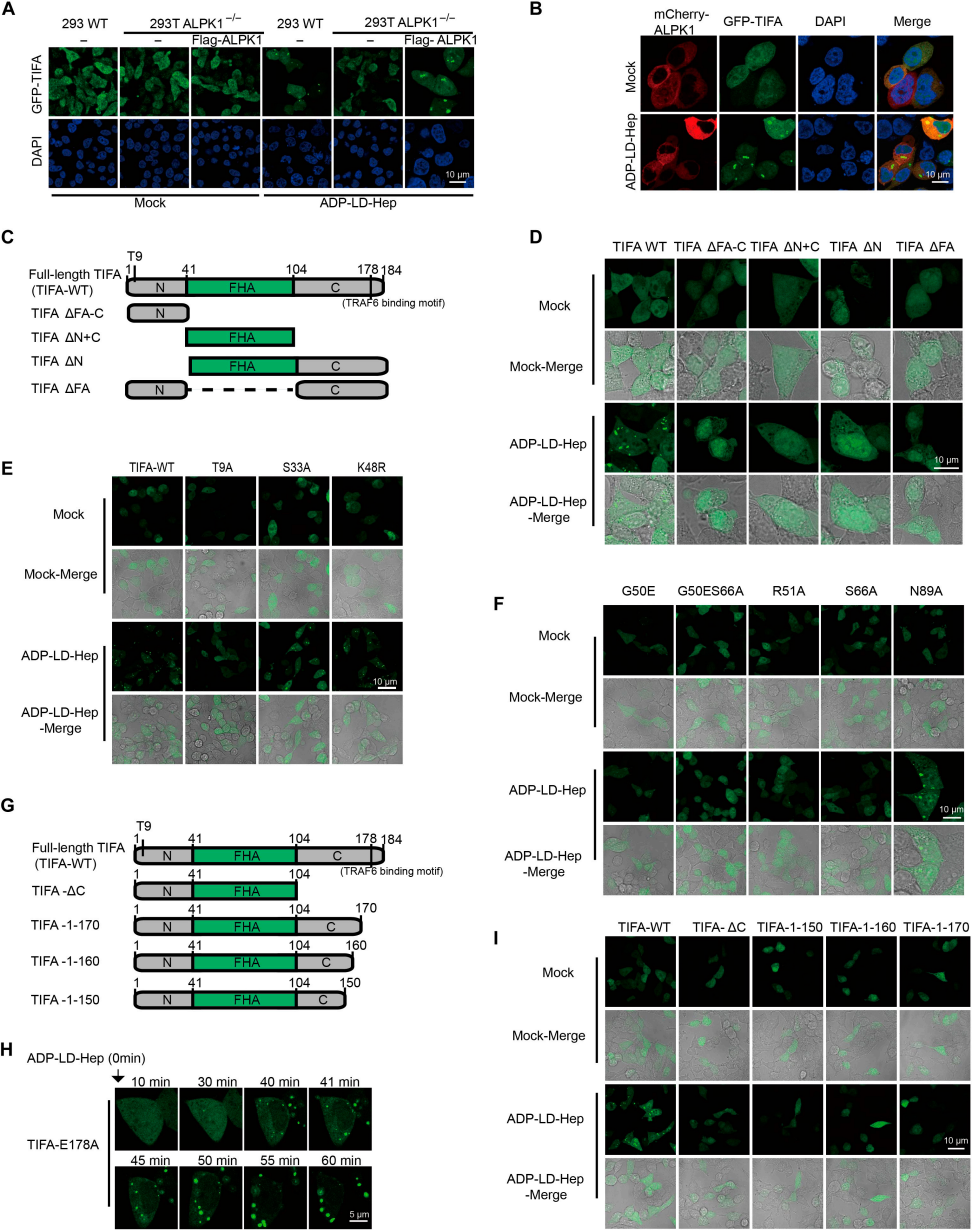
ALPK1, the pT9-FHA domain, and the IDR segment are necessary for TIFA liquid condensation. (A) Confocal images with DAPI-stained nuclei. GFP-TIFA was expressed in wild-type or ALPK1−/−293T cells expressing Flag-ALPK1 treated with ADP-Hep (10 μM) for 6 h, then the cells were fixed and observed under a NIKON AIR confocal microscope. Scale bars, 10 μm. (B) Confocal images with DAPI-stained nuclei. GFP-TIFA and mCherry-ALPK1 in 293T cells treated with ADP-Hep (10 μM) for 6 h. Scale bars, 10 μm. (C and G) Schematic diagrams showing the structure of TIFA and its truncation mutants. (D and I) 293T cells were transfected with TIFA or truncation constructs, and were treated with ADP-Hep (10 μM) for 6 h, followed by the analysis of TIFA or truncation mutants condensates by fluorescence microscopy. Scale bars, 10 μm. (E and F) Immunofluorescence analysis of TIFA and its mutants in HEK 293T cells. Scale bars, 10 μm. (H) Representative live cell images of GFP-TIFA-E178A condensates in 293T cells treated with ADP-Hep (10 μM). Scale bars, 5 μm. Data are representative of at least 2 independent experiments.

Considering that ALPK1 can directly bind to ADP-heptose and subsequently phosphorylate TIFA via its kinase domain [6], the role of ALPK1 in the formation of TIFA liquid condensation was investigated. Using CRISPR/Cas9 technology, an ALPK1-deficient 293T cell line was engineered, confirming that TIFA LLPS was completely prevented in knockout cells even when stimulated with ADP-heptose (Fig. 2A and Fig. S2A and B). Interestingly, rescued cells transfected with mCherry-ALPK1 were able to exhibit TIFA LLPS following stimulation with ADP-heptose (Fig. 2A and Fig. S2A and B). However, co-transfection experiments assessing immunofluorescence did not exhibit any colocalization of ALPK1 and TIFA, suggesting that ALPK1 cannot drive TIFA LLPS through multivalent interactions (Fig. 2B). These findings demonstrate that ALPK1 is a vital "switch" controlling the occurrence of TIFA LLPS in reaction to ADP-heptose stimulation.

The pThr9-FHA domain is a critical feature necessary for TIFA dimerization. The crystal structure determination of TIFA indicates that the model is antiparallel pairs of head-to-tail binding between pT9 and the FHA domain [22]. Dimerization of protein via weak polyvalent interactions is an intrinsic characteristic of liquid phase separation [23,24]. To investigate whether multivalent interactions also drive TIFA phase separation via the pThr9-FHA domain binding with TIFA dimers, 4 truncated constructs were engineered: a ΔFA-C truncation with a deletion of the FHA-C region, a ΔN+C truncation with a deletion of the N+C region, a ΔN truncation with a deletion of the N region, and a ΔFA truncation with a deletion of the FHA region (Fig. 2C). However, almost no liquid condensate was observed in cells overexpressing these truncations (Fig. 2D). Next, several site-specific mutants were constructed, including single mutants Thr9Ala (T9A), Ser33Ala (S33A), Lys48Ala (K48R), Gly50Glu (G50E), Arg51Ala (R51A), Ser66Ala (S66A), and Asn89Ala (N89A), and a double mutant G50E/S66A, which are critical for pThr9-FHA domain binding [14,22,23]. Various mutants were expressed in 293T cells, and immunofluorescence was employed to assess their performance (Fig. S2C and D). Most mutants of TIFA failed to undergo phase separation, except for N89A (Fig. 2E and F). TIFA mutant T9A phase separation was reconstituted in vitro to validate these cellular results. Purified T9A protein (Fig. S1D and E) was diluted in the presence of cell extracts treated with ADP-Hep and overexpressing ALPK1, and no formation of aggregates or droplets was observed even in the presence of ALPK1 (Fig. 1F). These results suggest that the pT9-FHA domain and its weak multivalent interactions are indispensable for the phase transition of TIFA.

The C-terminal region of TIFA contains a TRAF-binding motif, but its other functions have not been extensively examined. To investigate whether this region influences TIFA phase separation, we engineered a ΔC truncation with a deletion of the 104 to 184 region and overexpressed it in 293T cells. As expected, no liquid condensation was identified in these cells (Fig. 2G and I). To further probe the influence of the E178 residue, the TRAF6 binding site in TIFA, we transfected 293T cells with GFP-TIFA-E178A constructs. Following ADP-LD-Hep stimulation, phase separation of TIFA-E178A cells was apparent (Fig. 2H and Fig. S2E), indicating that the C-terminus of TIFA plays a pivotal role in TIFA phase transition, independent of the TRAF6 binding site. The investigation of Pfam and IUPred predicted that the C-terminal region of TIFA (human 170 to 184) was disordered (Fig. S2F), aligned with a previous x-ray diffraction model from Htiti (2.60Å; 5ZUJ) [22,23,25,26]. Moreover, we designed another 3 deletion constructs to assess the importance of the intrinsically disordered region (IDR) (150 to 184, 160 to 184, and 170 to 184). These truncated proteins exhibited diffused distribution, and no droplets were observed (Fig. 2G and I), suggesting that the IDR is required for liquid condensation. These data indicate that phosphorylation by the ALPK1 kinase domain, dimerization via the pThr9-FHA domain, and the IDR are all crucial for TIFA LLPS.

### The phenomenon of TRAF6 undergoing phase separation is universal

**Fig. 3.**
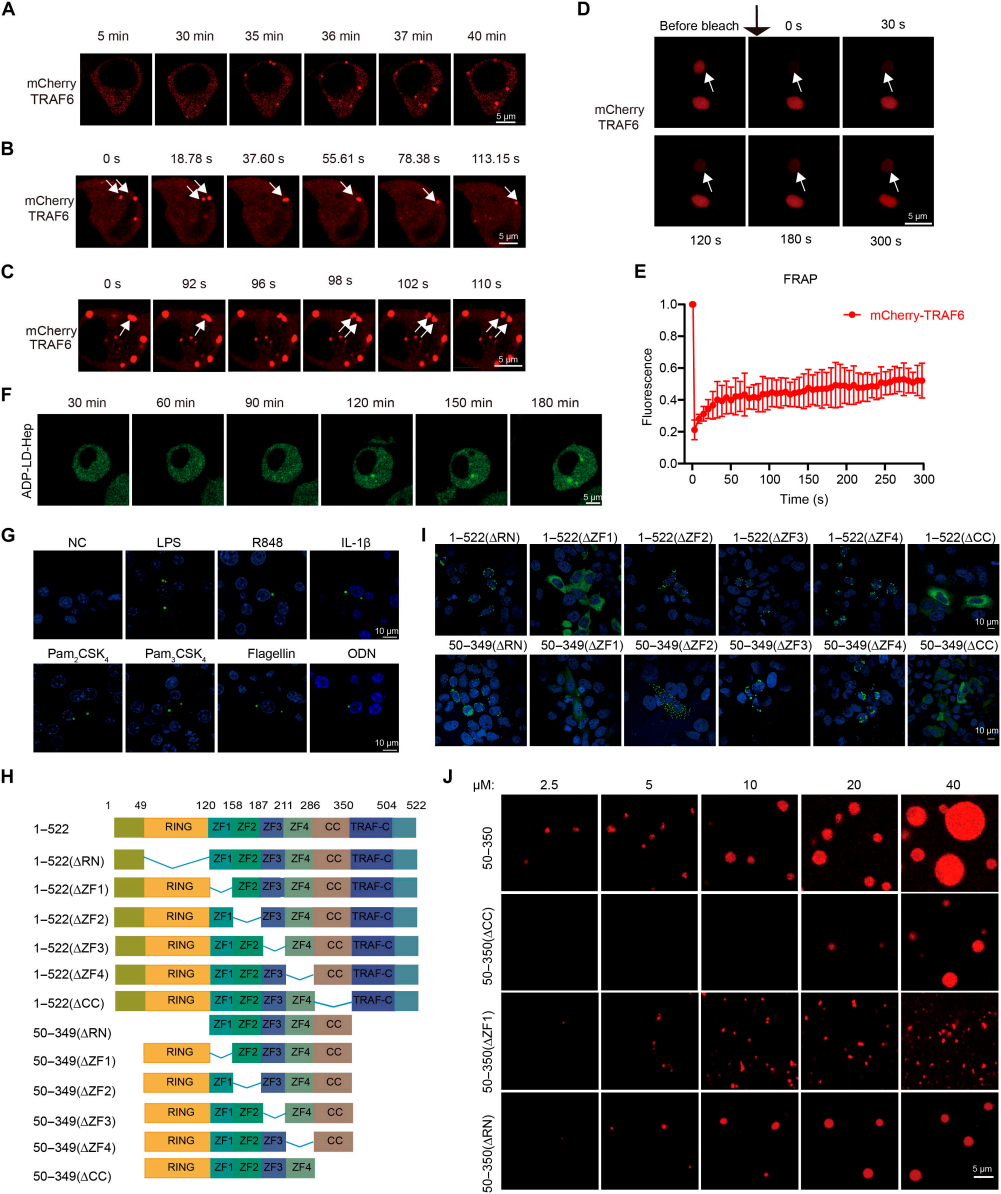
The phenomenon of TRAF6 undergoing phase separation is universal. (A) Representative live cell images of TRAF6 condensates in 293T cells stimulated with ADP-LD-Hep (10 μM) for 1 h. Scale bars, 10 μm. (B) Time-lapse micrographs of TRAF6 condensates formation and fusion (time 0 represents 60 min after ADP-LD-Hep treatment). Fusion events were observed in all 3 fields examined. (C) Time-lapse micrographs of the fission of TRAF6 condensates (time 0 represents 60 min after ADP-LD-Hep treatment). (D) FRAP analysis of TRAF6 puncta. Time 0 indicates the time at which the photo bleaching pulse was applied. Scale bars, 5 μm. *n* = 6 independent experiments. (E) Statistical analysis of FRAP of TRAF6 puncta over a time course of 300 s. (F) Representative live cell images of TRAF6 condensates in Raw264.7^EGFP-TRAF6^ cells stimulated with ADP-LD-Hep (10 μM) for 4 h. Scale bars, 5 μm. (G) Stimulation of Raw264.7^EGFP-TRAF6^ cells with LPS (100 ng/ml), R848 (2 μg/ml), IL-1β(100 ng/ml), Pam_2_CSK_4_ (100 ng/ml), Pam_3_CSK_4_ (100 ng/ml), Flagellin (100 ng/ml), and ODN (2 μg/ml) for 12 h, then the cells were fixed with 4% paraformaldehyde and observed under a NIKON AIR confocal microscope. (H) Schematic diagram of TRAF6 deletions and truncations. (I) Representative images of TRAF6 deletions and truncations overexpressed in HeLa cells. The cells were transfected with indicated plasmids, fixed after 24 h later. (J) Representative in vitro phase separation images of TRAF6(50–350), TRAF6(∆CC), TRAF6(∆ZF1), and TRAF6(∆RING) at different concentrations. Data information: Scale bars, 10 μm in (G) and (I) and 5 μm in (J).

TRAF6 serves as a central molecular hub, orchestrating interactions between upstream and downstream molecules in various signaling pathways, including TLR (Toll-like receptor), TCR (T cell receptor), RANK (receptor activator of nuclear factor kappa-B), IL-17R (interleukin-17 receptor), and TGFβR-I (transforming growth factor-beta receptor type I) signaling [27]. Previous studies have demonstrated that NEMO condensates encompass a multitude of proteins, such as TRAF6 and the TAK1 kinase complex, upon IL-1β stimulation. Additionally, evidence supports the notion that TRAF6 undergoes phase separation in response to LPS stimulation [28]. Building on these findings, we posited that TRAF6 experiences dynamic oligomerization during ALPK1/TIFA/TRAF6 activation.We overexpressed mCherry-tagged TRAF6 constructs in 293T cells to explore this hypothesis. Within 30 min of ADP-LD-Hep stimulation, TRAF6 droplets were observed, displaying dynamic changes in both size and number (Fig. 3A and Fig. S3A). Time-lapse microscopy unveiled the fusion and relaxation of TRAF6 droplets into larger ones (Fig. 3B). Furthermore, our observations captured TRAF6 droplets actively participating in the speck fission process (Fig. 3C). To assess the fluidity of these droplets, FRAP experiments were conducted. The results revealed a fluorescence recovery ranging from 20% to 60% within 5 min (Fig. 3D and E), suggesting that TRAF6 droplets are dynamic entities, exchanging with the environment—a characteristic feature of molecules undergoing phase separation.

Given the convergence of different TLRs and IL-1R signaling pathways toward TRAF6, we questioned whether TRAF6 exhibits a universal LLPS mechanism in signal transduction. To delve deeper into this under physiological conditions, we established a Raw 264.7 cell line (Raw264.7^EGFP-TRAF6^) stably expressing low levels of enhanced green fluorescent protein (EGFP)-TRAF6 (Fig. S3B). In a quiescent state, EGFP-TRAF6 displayed a diffused cellular distribution in Raw264.7^EGFP-TRAF6^ cells (Fig. 3F and G). However, upon stimulation with ADP-Hep or agonists of TLRs or IL-1R, such as LPS (TLR4), R848 (TLR7/8), Pam_2_CSK_4_ (TLR2/6), Pam_3_CSK_4_ (TLR1/2), Flagellin (TLR5), ODN (TLR9), and IL-1β (IL-1R), EGFP-TRAF6 underwent condensation into droplets in the cytosol (Fig. 3F and G). The droplets exhibited sizes in the range of micrometers (Fig. S3C). These findings indicate that the formation of TRAF6 droplets is regulated by upstream signals under physiological conditions, demonstrating the ability of TRAF6 to undergo phase separation into condensates at the cellular level under various conditions.

To further dissect the domains required for TRAF6 droplet formation, we designed a series of EGFP-tagged TRAF6 truncation and deletion plasmids based on published structures and ALFA FOLD 2 predictions, which were then overexpressed in HeLa cells (Fig. S3D). Neither the N-terminal 49 amino acids nor the TRAF-C domain proved necessary for TRAF6 condensate formation (Fig. S3D). Moreover, none of the individual domains, including the RING (really interesting new gene) domain, ZF (zinc finger) 1 to ZF4 domain, the coiled-coil domain, and the TRAF-C domain alone, were capable of forming condensates in cells (Fig. S3D). Intriguingly, TRAF6(50–349) proteins generated numerous droplets comparable to those formed by full-length TRAF6 (Fig. S3D). These data suggest that a combination of the RING domain, ZF1–ZF4, and the coiled-coil domain is sufficient for TRAF6 condensation.

To identify the essential domain for TRAF6 phase separation, deletion plasmids encompassing amino acids 50 to 349 were generated and overexpressed in HeLa cells (Fig. 3H). The results indicated that deleting ZF2, ZF3, or ZF4 separately had no significant effect on droplet formation for both full-length TRAF6 and TRAF6(50–349) (Fig. 3I). However, deleting either ZF1 or the coiled-coil domain strongly impaired droplet formation (Fig. 3I). The deletion of RING in full-length TRAF6 or TRAF6(50–349) led to protein aggregates in the nucleus. These findings suggest that both ZF1 and the coiled-coil domain are crucial for inducing condensate formation. A previous study has reported that the coiled-coil domain is responsible for TRAF6 oligomerization and constant interaction with Ubc13/UBE2N [29]. Here, we demonstrate that the coiled-coil domain plays an important role in encouraging TRAF6 phase separation.

To validate our cellular results, we reconstituted TRAF6 phase separation in vitro. Purified TRAF6(50–350), TRAF6(50–350 ∆CC), TRAF6(50–350 ∆ZF1), and TRAF6(50–350 ∆RN) proteins (Fig. S3E) were diluted in the reconstitution buffer (40 mM Tris–HCl [pH 7.5], 5 mM MgCl_2_, 150 mM NaCl, 10% glycerol, and 1 mM DTT) at different concentrations. Droplets appeared at 2.5 μM for TRAF6(50–350) proteins (Fig. 3J). The sizes of liquid droplets increased with the rising protein concentrations, indicating that TRAF6 phase separation occurs in a concentration-dependent manner. For TRAF6(50–350 ∆CC) proteins, smaller droplets only appeared above a concentration of 20 μM, underscoring the importance of the coiled-coil domain in lowering the threshold concentration for phase separation. In comparison to TRAF6(50–350) proteins, both TRAF6(50–350 ∆ZF1) and TRAF6(50–350 ∆RN) proteins generated smaller condensates at the same concentrations (Fig. 3J), suggesting that deleting either ZF1 or the RING domain weakens TRAF6’s ability to phase separate. The different capacities of TRAF6(50–350 ∆RN) proteins to form condensates in cells and in vitro may be attributed to its robust interaction with endogenous TRAF6 through coiled-coil domains. Given that the RING domain, ZF1, and coiled-coil domain are crucial for TRAF6 self-interaction, we speculate that the multivalent interactions of TRAF6 induce its phase transition. FRAP data showed that TRAF6(50–350) formed condensates with low mobility (Fig. S3F and G). Together, both cellular and in vitro results consistently demonstrate that TRAF6 undergoes phase separation during ADP-LD-Hep or other physiological stimulations. Phase separation of TRAF6 is a common mode in response to PAMPs, and the RING, ZF1, and coiled-coil domains play a fundamental role.

### TRAF6 is recruited in the TIFA condensates and promotes K63 polyubiquitin chain synthesis

**Fig. 4.**
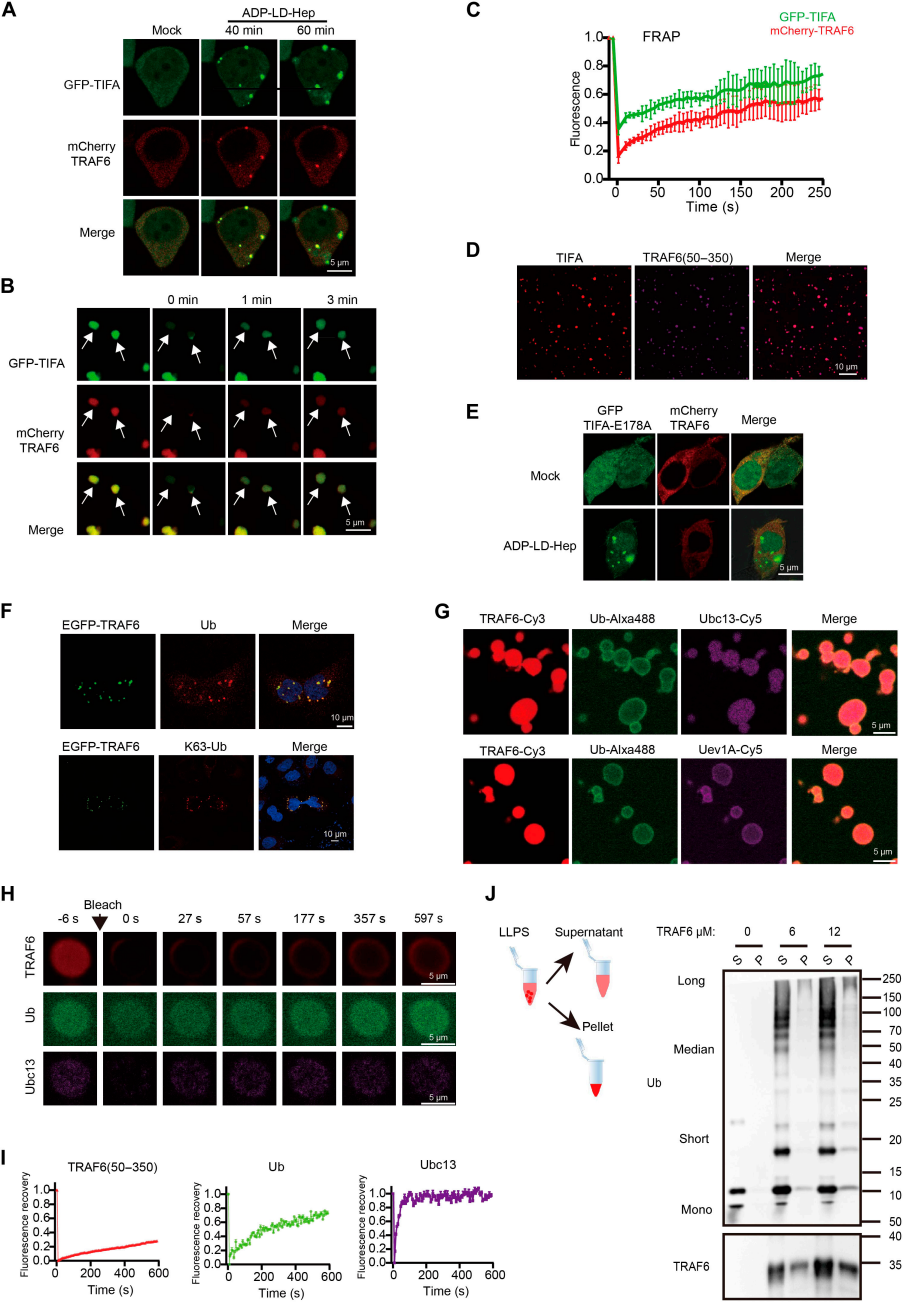
TRAF6 is recruited in the TIFA condensates and promotes K63 polyubiquitin chain synthesis. (A) Representative images of TIFA and TRAF6 condensates in 293T cells stimulated with ADP-LD-Hep (10 μM). Scale bars, 5 μm. (B) FRAP analysis of TIFA and TRAF6 puncta. Time 0 indicates the time at which the photo bleaching pulse was applied. Scale bars, 5 μm. *n* = 6 independent experiments. (C) Statistical analysis of FRAP of TIFA and TRAF6 puncta over a time course of 250 s. (D) Colocalization of TIFA-TRAF6 in vitro. Scale bars, 10 μm. (E) Colocalization of GFP-TIFA-E178A and mCherry-TRAF6 in 293T cells treated with or without ADP-LD-Hep (10 μM). Scale bars, 5 μm. (F) Upper panel, immunostaining of ubiquitin in EGFP-TRAF6 overexpressed HeLa cells; lower panel, immunostaining of K63 ubiquitin in EGFP-TRAF6 overexpressed HeLa cells. Scale bars, 10 μm. (G) Upper panel, in vitro LLPS images of TRAF6(50–350), ubiquitin and Ubc13; lower panel, *in vitro* LLPS images of TRAF6(50–350), ubiquitin and Uev1A. Scale bars, 5 μm. (H) Representative images of fluorescence recovery of a TRAF6(50–350), Ubc13, and Ub condensate. Scale bars, 5 μm. (I) Statistical analysis of the exchange kinetics of each protein in the TRAF6(50–350) condensates with solution in (H). The concentrations of TRAF6(50–350), Ubc13, Uev1A, Ub, and E1 were 20 μM, 1 μM, 1 μM, 50 μM, and 0.1 μM separately in (G) to (I). ATP concentration was 2 mM in (G) to (I). (J) Sedimentation assays to analyze the retention of ubiquitin chains after LLPS and ubiquitin chains synthesis. The concentrations of Ubc13, Uev1A, Ub, E1, and ATP were the same with that in (G). Data information: Scale bars, 5 μm in (G) and (H).

To elucidate whether TRAF6 is recruited in the ALPK1/TIFA signaling pathway during ADP-LD-Hep recognition, live cell imaging of TIFA and TRAF6 was performed in 293T cells. Following ADP-LD-Hep stimulation, TRAF6 puncta (red) colocalized with TIFA (green), forming 6 to 7 puncta per cell, and undergoing dynamic changes in both size and number (Fig. 4A and Fig. S4A). No significant difference was observed in the number of droplets formed by TIFA and TRAF6, respectively (Fig. S4B). The FRAP assay revealed that the fluorescence of mCherry-tagged TRAF6 and GFP-tagged TIFA rapidly recovered within 3 min after photobleaching (Fig. 4B and C). Furthermore, in vitro phase separation of TIFA and TRAF6(50–350) demonstrated significant colocalization (Fig. 4D). The results indicate that TIFA-TRAF6 droplets are dynamic and responsive to changes in the environment, typical of molecules undergoing phase separation.

To explore whether TRAF6 is recruited by TIFA through LLPS in the ALPK1/TIFA signaling pathway, 293T cells were co-transfected with mutant GFP-TIFA-E178A and mCherry-TRAF6 constructs, followed by stimulation with ADP-LD-Hep. As expected, phase separation of TIFA was still observed in the resulting cells, while TRAF6 was not detected in punctate structures (Fig. 4E and Fig. S4C). This suggests that the LLPS formation of TRAF6 depends on TIFA, specifically its E178 residue, which serves as the binding site for TRAF6 during LLPS. These findings provide evidence that TRAF6 is recruited in TIFA condensates in the ALPK1/TIFA signaling pathway in response to ADP-LD-Hep recognition.

TRAF6, functioning as an E3 ubiquitin ligase, orchestrates the synthesis and transfer of K63 ubiquitin chains in collaboration with the ubiquitin-activating enzyme (E1), the Ubc13-Uev1A (UBE2N/UBE2V1) ubiquitin-conjugating enzyme 2 (E2) complex, and Ub. The formation of LLPS of TIFA and TRAF6 has been substantiated. Our hypothesis posits that TRAF6 condensates harbor ubiquitin chains, a conjecture validated by immunofluorescence staining for Ub and K63 Ub in HeLa cells overexpressing EGFP-TRAF6. Notably, Ub and K63 Ub signals exhibited pronounced enrichment at TRAF6 condensates (Fig. 4F), signifying that LLPS facilitates the synthesis of K63 Ub chains.

To further validate this hypothesis, we reconstituted K63 Ub synthesis in vitro using purified proteins, including TRAF6(50–350), E1, Ubc13/UBE2N, Uev1A/UBE2V1, and Ub (Fig. S4D), within a buffer containing ATP. Immunoblotting against Ub demonstrated the successful synthesis of polyubiquitin chains in this in vitro system (Fig. S4E). Visualization of Alexa488-labeled Ub, Cy5-labeled Ubc13/UBE2N, and Cy3-labeled TRAF6(50–350) in the reconstitution system revealed the recruitment of both Ub and Ubc13/UBE2N into TRAF6 droplets (Fig. 4G). Similarly, Uev1A/UBE2V1 was also enriched in TRAF6 condensates (Fig. 4G). The results indicate that components involved in Ub synthesis are recruited into TRAF6 condensates upon LLPS. In FRAP experiments, TRAF6(50–350) fluorescence partially recovered 10 min after bleaching, indicating the formation of gel-like condensates. However, the fluorescent signals of Ubc13/UBE2N, Uev1A/UBE2V1, and Ub were almost completely restored within 10 min after bleaching (Fig. 4H and I and Fig. S4F and G). These data suggest that TRAF6, Ubc13, Uev1A, and Ub condensates exhibit dynamic exchange with the environment but at different rates.

Collectively, these findings suggest that TRAF6 is recruited within the TIFA condensates, forming low-fluidity condensates resembling scaffolds. In contrast, ubiquitin chain synthesis components can move and interact freely into and out of condensates, thereby accelerating the reaction. Our hypothesis posits that TRAF6 phase separation could foster K63 Ub chain synthesis. Indeed, after centrifugation of the in vitro ubiquitin system, short and median (2 to 8) ubiquitin chains were predominantly found in the supernatant, while long polyubiquitin chains were trapped in pellets consisting of TRAF6 condensates (Fig. 4J). Unanchored long polyubiquitin chains have been reported to be crucial for the phosphorylation and activation of TAK1. Moreover, TAB2 and TAB3 activate the NF-κB signaling pathway by binding to polyubiquitin chains. Thus, the LLPS of TIFA-TRAF6 and subsequent polyubiquitin chain synthesis may provide a platform for efficient ubiquitination and phosphorylation in the ALPK1/TIFA/TRAF6 signaling pathway.

### TIFA and TRAF6 phase separation recruits downstream proteins

**Fig. 5.**
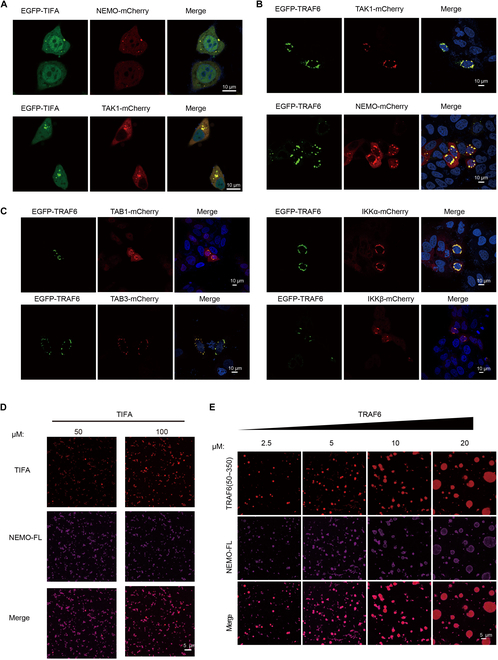
TIFA and TRAF6 phase separation recruits downstream proteins. (A) Representative images of EGFP-TIFA co-expressed with TAK1-mCherry or NEMO-mCherry separately in HeLa cells. (B and C) Representative images of EGFP-TRAF6 co-expressed with TAK1-mCherry, TAB1-mCherry, TAB3-mCherry, IKKα-mCherry, IKKβ-mCherry, and NEMO-mCherry separately. (D) Representative colocalization images of TIFA and NEMO in vitro at indicated concentrations. (E) Representative colocalization images of TRAF6 and NEMO in vitro at indicated concentrations. The concentration of NEMO in (D) is 10 μM. The concentration of NEMO in (E) is 20 μM. Scale bars, 10 μm in (A) to (C) and 5 μm in (D) and (E).

It is established that following TRAF6 activation and polyubiquitin synthesis, a cascade of proteins is triggered, culminating in the activation of NF-κB (nuclear factor-kappa-B), JNK (c-Jun N-terminal kinase), and p38 pathways. To scrutinize the participation of downstream proteins in TIFA-TRAF6 condensates, we individually overexpressed mCherry-tagged TAK1 and NEMO proteins either alone or together with EGFP-TIFA, in HeLa cells or 293T cells. The results unequivocally demonstrated the colocalization of these proteins with EGFP-TIFA condensates (Fig. 5A and Fig. S5A). Concurrently, we co-overexpressed mCherry-tagged TAK1, TAB3, IKKα, IKKβ, and NEMO proteins alongside EGFP-TRAF6 in HeLa cells. The results also showcased the colocalization of these proteins with EGFP-TRAF6 condensates (Fig. 5B and C). Notably, when TIFA was overexpressed alone or co-expressed with TRAF6, forming dispersed condensates in the cytoplasm upon ADP-Hep treatment (Figs. 1A, 2D, and 4A), the introduction of TAK1, TAB1, IKKα, and IKKβ led to the appearance of perinuclear condensates. This intriguing observation prompts speculation that, as TAK1, TAB1, IKKα, and IKKβ are more downstream in this signal pathway compared to TIFA, their proximity to the nucleus could facilitate the efficient transduction of signals into the nucleus.

Following that, within our in vitro reconstitution system, purified NEMO was present in TIFA condensates (Fig. 5D and Fig. S5B). The in vitro phase separation of TRAF6 and NEMO demonstrated that the fluorescent signal of NEMO increased within TRAF6 condensates as the concentrations escalated (Fig. 5E). Further insights were gained by employing purified TRAF6 and TAB1 peptides, revealing that TAB1 peptides were detected in TRAF6 condensates at concentrations exceeding 5 μM (Fig. S5C). These findings collectively establish that TIFA-TRAF6 phase separation has the capability to recruit downstream proteins into these condensates. Recent reports have highlighted that NEMO undergoes liquid phase separation induced by polyubiquitin chains [19]. Given that, in the process of ADP-Heptose-induced NF-κB activation, TIFA-TRAF6 precedes NEMO and is responsible for polyubiquitin chain production, and we posit that both the TIFA-TRAF6 complex and its product, polyubiquitin chains, contribute to promoting NEMO phase separation. TIFA, in this context, may serve as the driving force initiating the phase separation of downstream signaling molecules.

### TIFA LLPS promotes the activation of the ALPK1/TIFA signaling pathway

**Fig. 6.**
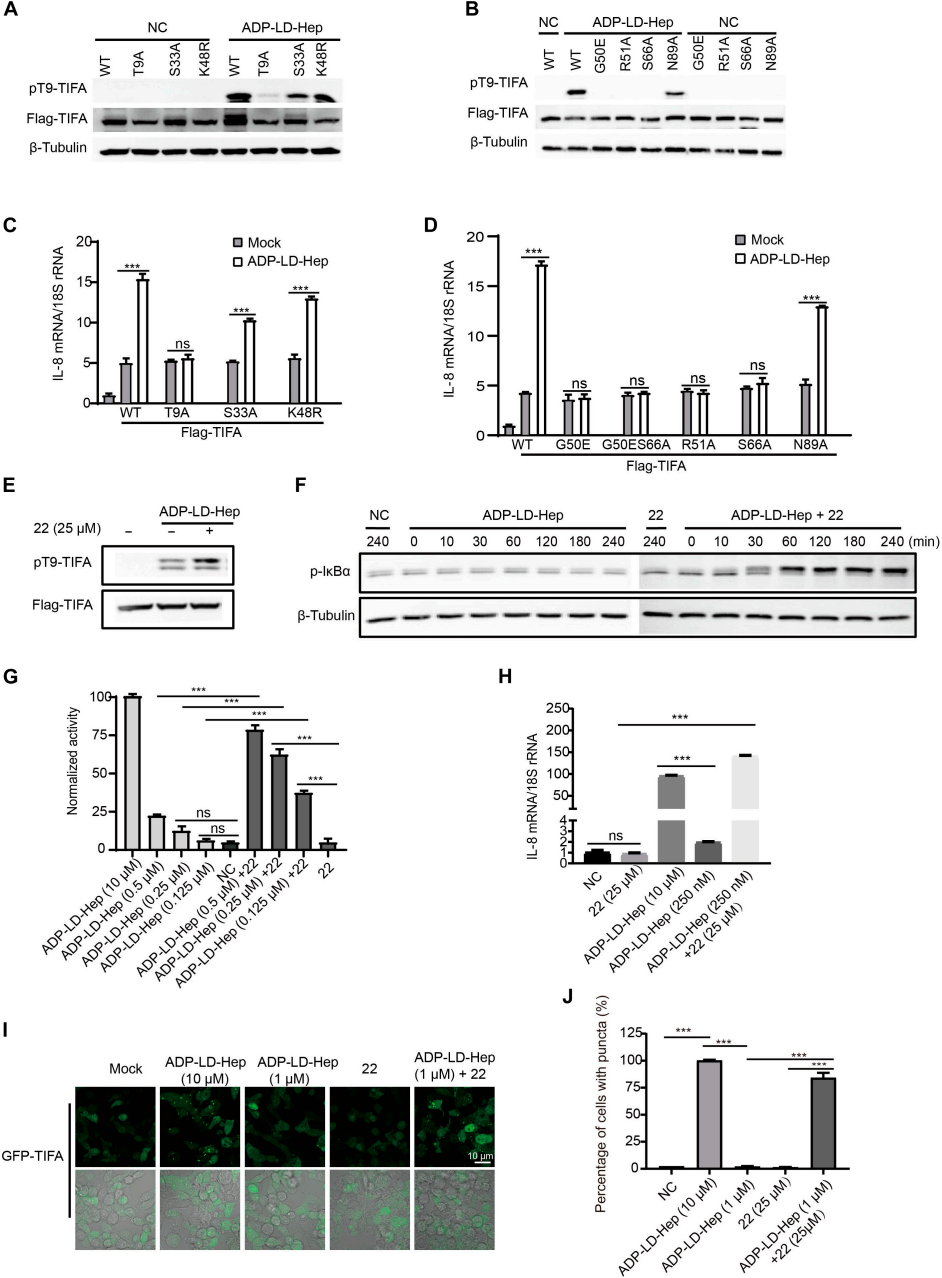
TIFA LLPS promotes the activation of the ALPK1/TIFA signaling pathway. (A and B) HEK293T cells were transfected with Flag-TIFA or Flag-TIFA mutants (T9A, S33A, K48R, G50E, R51A, S66A, or N89A) and treated with ADP-LD-Hep (10 μM) for 3 h before Western blot analysis. (C and D) TIFA or TIFA mutants (T9A, S33A, K48R, G50E, R51A, S66A, or N89A) overexpressed in 293T^TIFA KD^ cells were stimulated with ADP-LD-Hep (10 μM) for 24 h before RT-qPCR analysis of IL-8 mRNA. (E) Wild-type 293T cells expressing the indicated TIFA were treated with ADP-LD-Hep (2.5 μM) or ADP-LD-Hep (2.5 μM) and 22 (25 μM) for 3 h. TIFA phosphorylation was assessed by anti-pT9-TIFA immunoblotting. (F) Wild-type 293T cells expressing the indicated TIFA were treated with ADP-LD-Hep (2.5 μM) or ADP-LD-Hep (2.5 μM) and 22 (25 μM) for different times. IκBα phosphorylation was assessed by anti-IκBα immunoblotting. (G) Compound 22 (25 μM) synergistically activates ALPK1/TIFA signaling in the presence of ADP-LD-Hep (0.125 to 0.5 μM), while 22 alone was unable to induce any activation. Signals are normalized to the untreated cells. Data are mean ± SD; *n* = 3 independent experiments. (H) Compound 22 synergistically up-regulates the mRNA level of IL-8 in the presence of ADP-LD-Hep (0.25 μM). Data are means ± SD; the data shown are representative of at least 2 independent experiments. (I) Representative fluorescent images of TIFA liquid droplets induced by compound 22 (25μM) in the presence of ADP-LD-Hep (1 μM). Scale bars, 10 μm. (J) Quantification of TIFA puncta in different treatments. *n* = 3 independent experiments. Data are means ± SEM. Unpaired *t* test; n.s., not significant; ***P < 0.001.

Intrigued by the capacity of TIFA to undergo LLPS, we postulated that this mechanism might play a pivotal role in the ALPK1/TIFA signaling pathway. To scrutinize this hypothesis, we overexpressed FLAG-tagged TIFA and various mutants in 293T cells, evaluating their activation through immunoblotting. Interestingly, mutants K48R, S33A, and N89R of TIFA displayed successful phosphorylation at the T9 site, while other mutants failed to do so (Fig. 6A and B). To further corroborate these findings, we established a TIFA knockdown cell line (Fig. S6C). Notably, the induction of IL-8 expression by ADP-LD-Hep was nullified in the TIFA-KD cell line, a response rescued by wild-type TIFA, K48R, S33A, and N89R, but not by T9A and other mutants (Fig. 6C and D), aligning with previous immunofluorescence results. These results robustly support the assertion that TIFA’s phase separation is a crucial component of the ALPK1-TIFA-TRAF6 signaling pathway in response to ADP-LD-Hep recognition.

To further underscore the essential role of TIFA LLPS in activating the ALPK1-TIFA-TRAF6 signaling pathway, we conducted a chemical biology investigation. Following extensive screening using a HEK blue reporter cell-based assay, 5 compounds, including compounds 13, 22, 41, 73, and 256 (structures shown in Fig. S6D), were identified as activators of the ALPK1-TIFA-TRAF6 signaling pathway in the presence of low concentrations of ADP-LD-Hep. Subsequently, these 5 compounds were tested in HEK blue null cells in a dose-dependent manner, with adenosine monophosphate compound **22** chosen as a molecular probe (Fig. S6E). Intriguingly, TIFA phosphorylation was not induced by ADP-LD-Hep at a concentration of 2.5 μM, but the phosphorylation level was augmented upon the addition of compound **22** (25 μM) (Fig. 6E). Moreover, the co-presence of **22** (25 μM) and ADP-LD-Hep (0.5 μM) intensified the phosphorylation of IκBα protein compared to ADP-LD-Hep (0.5 μM) alone (Fig. 6F). NF-κB activation stimulated by ADP-LD-Hep in HEK blue null cells was dose-dependent (IC_50_ = 2.3 μM) (Fig. S6A and B). Minimal NF-κB activation was observed with ADP-LD-Hep at 0.25 μM, while treatment with ADP-LD-Hep (0.25 μM) and compound **22** (25 μM) synergistically enhanced NF-κB activation, almost reaching a level similar to ADP-LD-Hep at 10 μM (Fig. 6G). The results of immunoblotting and NF-κB activation were consistent with the expression of IL-8 (Fig. 6H). Hence, these findings suggest that compound **22** works synergistically with ADP-LD-Hep to activate TIFA and downstream signaling.

To further affirm the necessity of TIFA LLPS for the activation of the ALPK1-TIFA-TRAF6 signaling pathway induced by small-molecule chemicals, GFP-TIFA was overexpressed in 293T cells. It was observed that TIFA LLPS gradually diminished with a decreasing concentration of ADP-LD-Hep (Fig. S6F and G). When the concentration of ADP-LD-Hep was reduced to 1 μM, TIFA exhibited a diffuse distribution compared to the speck formation observed with ADP-LD-Hep at 10 μM (Fig. 6I). Intriguingly, TIFA liquid condensation was reinstated only by adding a mixture of ADP-LD-Hep (1 μM) and compound **22** (25 μM), rather than **22** alone (Fig. 6I). Immunofluorescence statistical analysis revealed that this mixture restored TIFA’s liquid-like state to around 70% of its original intensity compared to the intensity produced by ADP-LD-Hep at 10 μM (Fig. 6J). Therefore, our data suggest that compound **22** acts as a molecular probe that enhances the LLPS of TIFA through its regulatory effects. In summary, these results propose that TIFA-TRAF6 clusters in a liquid-like state as “microreactors” for recruiting downstream signaling molecules.

## Discussion

The adapter protein TIFA serves as the initial host factor in controlling inflammation following bacterial heptose recognition [15]. Upon stimulation with ADP-Heptose, TIFA orchestrates the oligomerization of TRAF6, leading to the subsequent activation of NF-κB. Moreover, the occurrence of TIFA large complexes (TIFAsomes) [12] has been observed during pathogenic infections through a TIFA-dependent cytosolic monitoring pathway. Existing evidence suggests that the formation of TIFAsomes is predominantly triggered by gram-negative bacterial infections, as gram-positive bacteria do not produce heptose [12]. However, the biochemical mechanisms underpinning TIFAsomes remain elusive, and the characteristics of these droplets, along with their implications in the ALPK1-TIFA-TRAF6 signaling pathway, are yet to be explored.

**Fig. 7.**
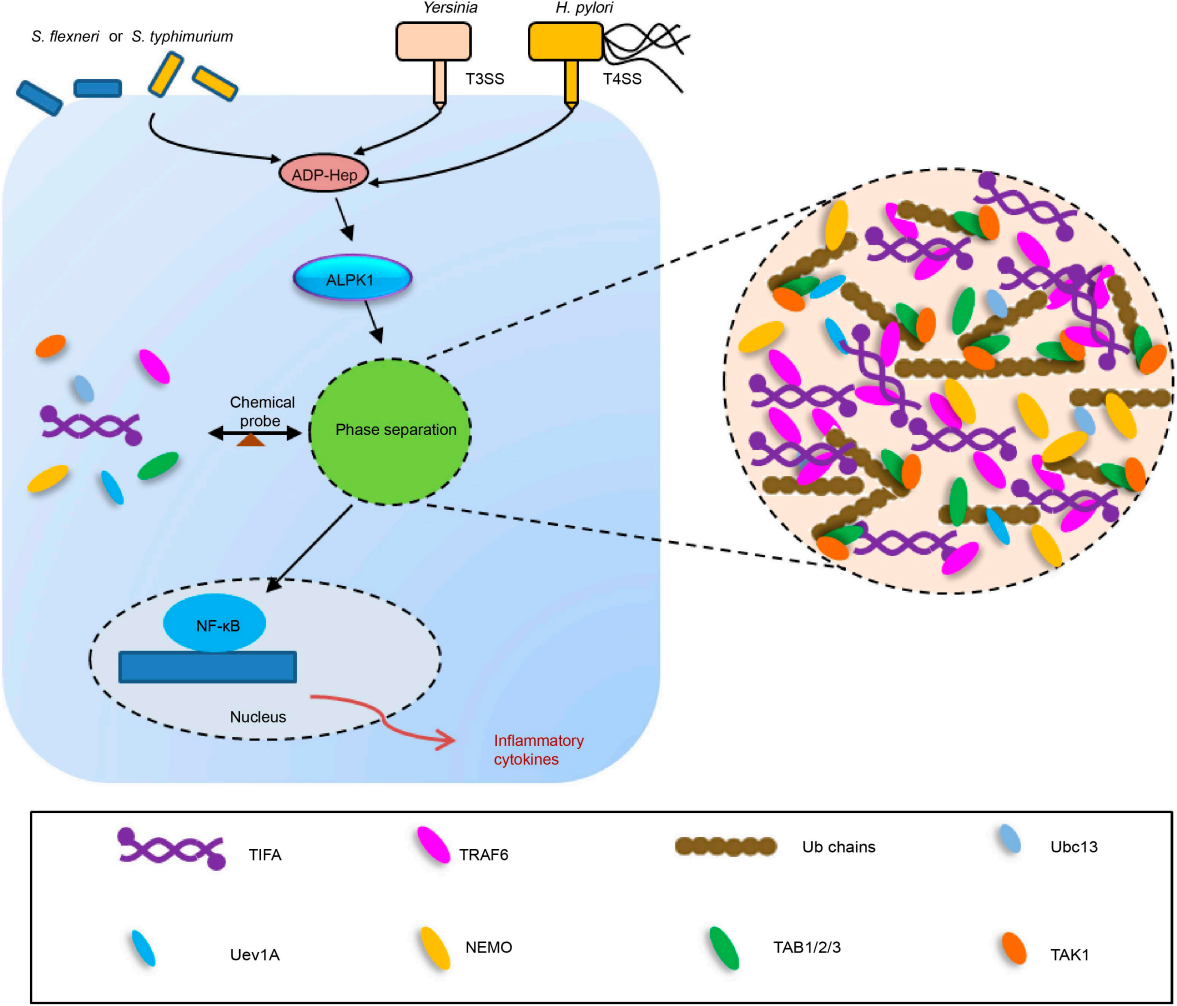
Proposed model of the ALPK1-TIFA-TRAF6 axis that drive their liquid phase separation.

In recent years, LLPS has emerged as a crucial mechanism that orchestrates physiological processes by modulating molecular concentrations and sequestering undesirable proteins [30–35]. Resident proteins of antiviral stress granules, such as the PRRs RIG-I (retinoic acid-inducible gene I) and MDA-5 (melanoma differentiation associated gene 5), exhibit liquid properties and undergo LLPS [36–39]. While numerous instances of "structured" supramolecular assemblies in innate immune responses exist [40], only a select few supermolecules have been demonstrated to undergo phase separation. Examples include the validation of signal molecular clustering in T cell receptor signal transduction through phase separation [41]. The phase transition induced by double-stranded DNA (dsDNA) in cyclic GMP-AMP synthase (cGAS) promotes the production of cyclic GMP-AMP (cGAMP), subsequently activating the stimulator of interferon gene (STING) and interferon type I [42]. Mutants of the tumor suppressor Neurofibromin 2 have been identified to form liquid biocondensates induced by both the RLR-MAVS pathway and the cGAS-STING pathway, containing activated IRF3, thereby suppressing nucleic acid sensing [43]. LLPS induced by the combination of polyUb with NEMO, serving as a microreactor, considerably enhances the activation of IKK, TAB1/2/3, and various phosphorylation and ubiquitination reactions [19]. In this study, we broaden our understanding of the role of phase separation in immunoregulation by unveiling TIFA-TRAF6 LLPS within the ALPK1-TIFA-TRAF6 pathway (Fig. 7).

In this investigation, we present compelling evidence supporting the occurrence of LLPS in TIFA induced by ADP-LD-Hep, challenging the previously widely accepted notion of complex TIFA structures. To gain deeper insights into the molecular mechanism governing TIFA liquid condensation, we explored the involvement of ALPK1. The results elucidate that ALPK1 serves as a “switch”, exerting control over the initiation of TIFA LLPS in response to ADP-heptose stimulation. LLPS, characterized by the accumulation of proteins through weak polyvalent interactions, inherently underlies this process. Our hypothesis posits that TIFA phase separation is propelled by these weak polyvalent interactions. To further dissect the molecular requirements for TIFA phase transition, we engineered truncated constructs and site-specific mutants, revealing the pivotal role of the pThr9-FHA domain and its weak multivalent interactions in driving TIFA phase transition. Additionally, we established that the C-terminal region of TIFA plays a crucial role in its phase transition, irrespective of the TRAF6 binding site. The IDR of TIFA was identified as an essential component for liquid condensation.

We propose that the LLPS of TIFA plays a crucial role in expanding the diversity of the ALPK1/TIFA/TRAF6 signaling pathway. Two key observations support our rationale. Firstly, we have demonstrated that TRAF6, a vital component in this pathway, also exhibits phase separation in response to ADP-Hep-induced inflammation. Notably, this phenomenon of TRAF6 undergoing phase separation is conserved across various inflammatory signal pathways. Secondly, our investigation reveals that TIFA itself undergoes LLPS and has the capacity to recruit downstream proteins. The collaboration between TIFA-TRAF6 and the formation of long polyubiquitin chains potentially establishes a “reaction factory”. This specialized microenvironment could facilitate more efficient phosphorylation and ubiquitination reactions, particularly at elevated concentrations triggered by ADP-LD-Hep or gram-negative bacterial stimulation.

Our primary goal was to elucidate the interplay between TIFA LLPS and the activation of downstream signaling pathways. Through a series of experiments involving TIFA overexpression and knockdown, we uncovered that the LLPS capability of TIFA exerts a marked influence on its functional role in the ALPK1/TIFA signaling pathway. Building on the wealth of research into the chemical and biological modulation of phase separation, we shifted our focus to the screening of small molecular chemical probes. Among these, compound **22** emerged as a promising candidate and was employed to activate ALPK1-TIFA-TRAF6 signaling at low concentrations of ADP-LD-Hep. Our findings further suggest that molecular probes can effectively regulate phase separation. As part of future endeavors, we are actively optimizing and assessing the biological efficacy of these molecular probes, aiming to develop the first immunomodulator capable of regulating the ALPK1-TIFA-TRAF6 signaling pathway.

To summarize, our study reveals that TIFA undergoes dynamic liquid-like phase separation rather than forming higher-order structures in response to ADP-LD-Hep induction. The LLPS of TIFA is primarily orchestrated by ALPK1, the pT9-FHA domain, and the IDR segment. Additionally, we found that TRAF6 undergoes phase separation during ADP-Hep-induced inflammation, and this phenomenon is universal across different inflammatory signal pathways. Moreover, TRAF6 is recruited within the TIFA condensates, where it promotes the synthesis of K63 polyubiquitin chains. The condensates facilitate the synthesis of long K63-linked polyubiquitin chains, leading to the recruitment, enrichment, and activation of downstream effectors and robust inflammatory signal transduction. In essence, TIFA serves as a sensor for upstream signals, initiating LLPS of itself and downstream proteins, forming TIFA-TRAF6 membraneless condensates in the ALPK1-TIFA-TRAF6 pathway. This newfound understanding of the TIFA-TRAF6 regulatory mechanism presents a promising strategy for the development of therapeutic biotechnology.

## Materials and Methods

The materials used in this work are listed in Supplementary Table S1.

### Cell culture, treatment, and transfection

HEK-Blue Null cells stably transfected with an NF-κB secreted embryonic alkaline phosphatase (SEAP) reporter was used to assess the potency and specificity of compounds. HEK 293T cells, HEK-Blue Null cells, HeLa cells, and RAW 264.7 cells were cultured in DMEM (Dulbecco's modified eagle medium) (Invitrogen) with 10% FBS (fetal bovine serum) (Invitrogen), 100 U/ml penicillin, and 100 μg/ml streptomycin. All cells were maintained at 37 °C in an atmosphere of 5% CO_2_. Cells were treated with TransSafe Mycoplasma Prevention Reagent (TransGen Biotech). Cells were transfected with plasmids using Lipofectamine 3000 according to the manufacturer’s instruction (Invitrogen).

### SEAP reporter assay

HEK-Blue Null cells (5× 10^4^ cells/well) were plated in a 96-well plate in DMEM and treated with 10 μM ADP-Heptose (J&K Scientific) and the indicated concentrations of the compounds. Cell culture medium was assayed with Quanti-Blue (InvivoGen) following the manufacturer’s recommendation after 24 h of incubation at 37 °C. The SEAP reporter was constructed as an interferon (IFN)-β promoter fused with 5 NF-κB and AP-1-binding sites. SEAP was quantified by measuring absorbance at 620 nm. Data were normalized as follows: ligand-treated cells are 100% activated and untreated cells are 0% activated. Each data point represents the mean and standard deviation of at least 3 biological replicates.

### Plasmid construction

Plasmid construction was carried out with commercial plasmid detailed in the previous description [44]. The human TIFA and mutants were cloned into pEGFPC1 for transfection and into pET22b- 6×His tag vector for protein purification. All constructs were confirmed by sequencing. The plasmids used in this work are listed in Table S1.

### Cell line construction

HEK 293T/17 cells were transfected with retroviral plasmids pQCXIP-EGFP-TRAF6 plus packaging plasmids pVSVG and pHIT and the virus particles were harvested 48 h after transfection, filtered through a 0.45 μm membrane filter and stored at −80 °C. RAW 264.7^EGFP-TRAF6^ stable cells were generated by infecting the RAW 264.7 with harvested retrovirus in the presence of 1 μg/ml polybrene. After 48 h of culture, transduced cells were selected with 2 μg/ml puromycin. The positive cells were verified by Western blotting and RT-qPCR (quantitative real-timepolymerase chain reaction) analyses. LPS (100 ng/ml), IL-1β (100 ng/ml), Pam_2_CSK_4_ (100 ng/ml), Pam_3_CSK_4_ (100 ng/ml), flagellin (100 ng/ml), R848 (2 μg/ml), and ODN (1 μM) were added to the DMEM without FBS for the indicated time.

pLKO.1 TIFA-shRNA (TRC_ID: TRCN0000134082, TRCN0000138828, and TRCN0000137712) and pLKO.1 was obtained to make stable knockdown cell lines from Center of Biomedical Analysis, Tsinghua University. HEK 293T/17 cells were transfected with the indicated lentivirus plasmids together with the packing plasmids psPAX2 and pMD2.G. The virus package, infection of 293T, and puromycin selection were similar to the generation of RAW 264.7^EGFP-TRAF6^ stable cells.

PLKO.1 ALPK1-sgRNA and CRISPR plasmids (8 μg) were constructed and used to generate KO cell lines. HEK293T/17 cells were transfected with the indicated lentiviral expression plasmid (8 μg) together with the packaging plasmids psPAX2 (6 μg) and pMD2.G (2 μg) in 10-cm dishes. At 24 and 48 h post-transfection, cell media containing lentiviral particles were harvested, sterile filtered, and subsequently used to infect the target cells in the presence of 8 μg/ml polybrene. Stably transduced cells were then selected using 5 μg/ml puromycin (InvivoGen) after 48 h of culture. The TIFA KO cell line was constructed in a similar way. The guide RNA (gRNA) sequences used for KO cell line generation were as follows:

*ALPK1*-g1: TCGGTCTGTATACAGATCAGAGG

*ALPK1*-g2: CAGAGACGTCGAGCCCATACAGG

*TIFA*-g1: TCCAGCGAAGTGGTGAAATT

*TIFA*-g2: CAGATGACGGTTTACCATCC

### Immunofluorescence

Cells were seeded in 4-well glass dishes and transfected with the plasmids. Lipofectamine 3000 were used for transfection. Twenty-four hours later, the supernatants were removed and the cells were washed with PBS. Next, the cells were fixed with 4% paraformaldehyde for about 20 min, infiltrated with Triton X-100 [0.25% (v/v)] for 10 min, then blocked with bovine serum albumin (BSA) [3% (w/v)] for 60 min. The cells were incubated with primary antibodies in 3% (w/v) BSA overnight at 4 °C or for 2 h at room temperature (RT). Subsequently, the cells were incubated for 60 min at RT using fluorescent secondary antibodies and DAPI (4′,6-diamidino-2-phenylindole). Finally, the cells were washed 3 times with PBS, and images were captured using a Nikon A1RMP Confocal microscopy (Nikon, Japan). Colocalization was evaluated using Imaris software.

### Protein expression and purification

The purification details of the following proteins were described in the previous work [45]. Recombinant TRAF6(50–350) proteins and other variants were expressed in the *E. coli* strain *Transseta* (DE3) transformed with corresponding plasmids. The *E. coli* transformed with corresponding TRAF6 plasmids were grown at 37 °C to an O.D. of 0.6–0.8, and then were induced with 0.2 mM IPTG (isopropyl β-D-thiogalactoside) and 0.1 mM ZnCl_2_ at 16 °C for 16 h. The bacteria were centrifuged and crushed under high pressure in the lysis buffer containing 25 mM Tris–HCl (pH 8.0), 150 mM NaCl, and 1 mM PMSF (phenylmethanesulfonyl fluoride). After centrifugation at 13,000*g* for 1 h, the supernatants were collected and loaded onto Ni-NTA Agarose column. The Agarose and column were washed with the lysis buffer with 20 to 50 mM imidazole. The TRAF6 proteins were eluted with the lysis buffer with 200 to 300 mM imidazole. The eluted proteins were concentrated and purified on AKTA with a Source Q column. The purity of proteins was verified by SDS-PAGE. TAB1(468–504) was expressed in the *E. coli* strain *Transseta* (DE3) transformed with pET-21a plasmid with 0.5 mM IPTG induction at 18 °C for 20 h. The eluted proteins were concentrated and the buffer was changed to 25 mM Tris–HCl (pH 8.0) and 150 mM NaCl by a desalting column. GST-NEMO was expressed in the *E. coli* strain *Transseta* (DE3) transformed with the pGEX-6p-1 plasmid encoding NEMO. The *E. coli* were induced with 0.5 mM IPTG at 18 °C for 20 h. The bacteria were centrifuged and crushed under high pressure in the lysis buffer containing 25 mM Tris–HCl (pH 8.0), 150 mM NaCl, and 1 mM PMSF. After centrifugation at 13,000*g* for 1 h, the supernatants were collected and loaded onto a Glutathione Sepharose 4B resin (GE Healthcare) column. Following washing with the lysis buffer, the GST tag was cleaved on column with homemade CST-HRV 3C protease at 4 °C overnight. Cleaved proteins were collected and concentrated. His_6_-MBP-SUMO-Ub was expressed in the *E. coli* strain *Transseta* (DE3) transformed with corresponding plasmids with 0.5 mM IPTG induction at 18 °C for 16 h. The purification procedure was the same as TRAF6. The His_6_-MBP-SUMO tag was removed by sumo protease at 4 °C overnight and the Ub proteins were further purified on AKTA equipped with a Source Q column. Ubc13, Uev1A, and UBE1 were expressed in the *E. coli* strain *BL21* (DE3) with 0.5 mM IPTG induction. The purification procedures were similar to TRAF6. After elution, His_6_-Ubc13, His_6_-Uev1A, and His_6_-UBE1 were concentrated and further purified by gel filtration. The gel filtration columns were equilibrated with 25 mM Tris (pH8.0), 150 mM NaCl, 5% glycerol, and 2 mM DTT (Superdex 75 10/300 for His_6_-Ubc13 and His_6_-Uev1A; Superdex 200 10/300 for His_6_-UBE1).

TIFA was expressed in the *E. coli* strain *Transseta* (DE3) transformed with pET-22b plasmid with 0.5 mM IPTG induction at 18 °C for 20 h. The bacteria were centrifuged and crushed under high pressure in the lysis buffer containing 25 mM Tris–HCl (pH 8.0), 300 mM NaCl, and 1 mM PMSF. After centrifugation at 13,000*g* for 1 h, the supernatants were collected and loaded onto Ni-NTA Agarose column. The Agarose and column were washed with the lysis buffer with 10 to 30 mM imidazole. The TIFA proteins were eluted with the lysis buffer with 200 to 300 mM imidazole. The eluted proteins were concentrated and purified on AKTA with a Source Q column and gel filtration chromatography (GE Healthcare Life Sciences). The purity of proteins was verified by SDS-PAGE.

### Sedimentation assay

After conducting the in vitro ubiquitination assay, TRAF6 droplets were spun down at 12,000*g* for 10 min. The amount of ubiquitin or polyubiquitin chain was analyzed by Western blot in the supernatant and pellet.

### RT-qPCR

For RT-qPCR analysis, the cells were inoculated onto a 6-well plate and treated as shown in the figure. Subsequently, total RNA was extracted by TRIzol. Reverse transcription was performed with iScript cDNA synthesis kit and analysis was performed with iTaq Universal SYBR Green Supermix in a Bio-Rad T100 thermal cycler. All reagents were used as stipulated in the manufacturer’s specifications. Internal controls were GAPDH (human). Please refer to Table S2 for primer sequences.

### Live-cell imaging

In the live-cell imaging experiments, HEK 293T cells were transfected with GFP-TIFA and mCherry-TRAF6 and grown on 4-well glass dishes to a suitable density. After 24 h, ADP-LD-Hep was introduced to the cells. Images were taken using a 100× oil objective Nikon A1 camera every 10 s, and were subsequently evaluated with the use of Imaris.

### Protein fluorescent labeling

TIFA protein (2.5 mg) was labeled with FITC (fluorescein isothiocyanate) using HOOK Dye Labeling Kit (G-Biosciences, #786-141) according to the manufacturer’s instructions.

TIFA, recombinant TRAF6(50–350), and other variants were labeled with Cyanine 3 (AAT Bioquest); Ubc13, Uev1A, NEMO, and TAB1(468–504) were labeled with Cyanine 5 (AAT Bioquest); and Ub was labeled with iFluor 488 (AAT Bioquest) according to the manufacturer’s instructions.

### LLPS in vitro

The purified TIFA or TIFA mutant T9A and Flag-ALPK1 cell lysate were incubated with 200 μM ATP, ADP-Heptose (10 μM), and 1× reaction buffer containing 25 mM Tris–HCl (pH 7.5), 150 mM NaCl, 5 mM MgCl_2_, and 1 mM dithiothreitol (DTT). The mixture was then prepared 30 min before imaging at RT by adding ADP-LD-Heptose. For imaging, protein solutions were loaded onto a glass-bottom 384-well plate, and images were taken using a Nikon A1R HD25 confocal microscope with a 100× oil-immersion objective.

The purified TRAF6(50–350) and derivatives, NEMO, and TAB1(468–504) (2% fluorescence labeled) were diluted to the indicated concentration in a buffer containing 40 mM Tris–HCl (pH 7.5), 5 mM MgCl_2_, 150 mM NaCl, 10% glycerol, and 1 mM DTT. In the ubiquitination system, 2 mM ATP, 50 μM Ub, 0.1 μM UBE1, 1 μM Ubc13, and 1 μM Uev1A were added to the mixtures. Mixtures were transferred to a 384-well glass bottle plate (cellvis) and images were captured by confocal microscopy.

### In vitro FRAP assays

In vitro FRAP assays were carried out as previously described with some adjustments [42]. In detail, FRAP measurements were carried out on a Nikon A1RMP confocal microscope (Nikon, Japan) at room temperature. TIFA spots of ~1 μm diameter were photo bleached with 10% laser power for 0.5 s using 488 nm. Time-lapse images were collected over a 10-min time course after bleaching at 6-s intervals. TRAF6(50–350), Ubc13, Uev1A, and Ub signals were photo bleached by similar procedures to that of TIFA.

### Quantification and statistical analysis

GraphPad Prism 7 was utilized for quantification and statistical analysis. All of the results were performed with at least triplicate samples as independent biological replicates. Student’s *t* test and 2-way analysis of variance (ANOVA) with Sidak’s multiple comparisons post-hoc test were used to determine the statistical significance A log-rank (Mantel-Cox) test was adopted for survival analysis. ***P < 0.001 was considered statistically significant.

### Western blotting analysis

To perform Western blotting analysis, cells were lysed with RIPA (radio immunoprecipitation assay) lysis buffer supplemented with protease inhibitor cocktail and a phosphatase inhibitor. After centrifuging at 12,000*g* for 10 min, the supernatants were collected to determine the protein concentration using bicinchoninic acid (BCA) assay. The protein samples were separated by SDS-PAGE, transferred onto polyvinylidene difluoride (PVDF) membranes, and blocked with 5% non-fat milk. The primary antibodies were added and incubated at 4 °C overnight, followed by incubation with secondary antibodies for 1 h at room temperature. The membranes were developed with a chemiluminescent substrate, and visualized using an iBright 1500 imaging system. The densitometric analysis was determined by ImageJ software.

## Data Availability

All data are available in the manuscript or the Supplementary Materials.
