# Supplementary material for: ADP-Hep-Induced Liquid Phase Condensation of TIFA-TRAF6 Activates ALPK1/TIFA-Dependent Innate Immune Responses
Source: Research (Wash D C). 2024 Feb 14;7:0315. doi: 10.34133/research.0315 (PMC10865109; doi:10.34133/research.0315)
Supplement: Supplementary 1 — Figs. S1 to S6 Tables S1 and S2 [file research.0315.f1.zip › 20240115-Supplementary Material.docx]

Supplementary Materials for

**ADP-Hep-Induced** **Liquid Phase Condensation of TIFA-TRAF6 Activates ALPK1/TIFA-Dependent** **Innate Immune Responses**

Liping Li^1,2,5^, Jia Wang^1,3,5*^, Xincheng Zhong^1,5^, Yaoyao Jiang^1^, Gaofeng Pei^1,4^, Xikang Yang^1^, Kaixiang Zhang^1^, Siqi Shen^1^, Xue Jin^1^, Gaoge Sun^1^, Chaofei Su^1^, Shuzhen Chen^2^, and Hang Yin^1*^

*Corresponding author: wangjia623@163.com; yin_hang@tsinghua.edu.cn (H. Y.)

**The PDF file includes:**

Fig. S1. TIFA forms liquid-like condensates stimulated with ADP-heptose.

Fig. S2. ALPK1, the pT9-FHA domain, and the IDR segment are necessary for TIFA liquid condensation

Fig. S3. The phenomenon of TRAF6 undergoing phase separation is universal.

Fig. S4. TRAF6 is recruited in the TIFA condensates and promotes K63 poly-ubiquitin chain synthesis.

Fig. S5. TIFA and TRAF6 phase separation recruits downstream proteins.

Fig. S6. TIFA LLPS promotes the activation of the ALPK1/TIFA signaling pathway.

Table S1. Materials.

Table S2. Primers used for quantitative PCR.


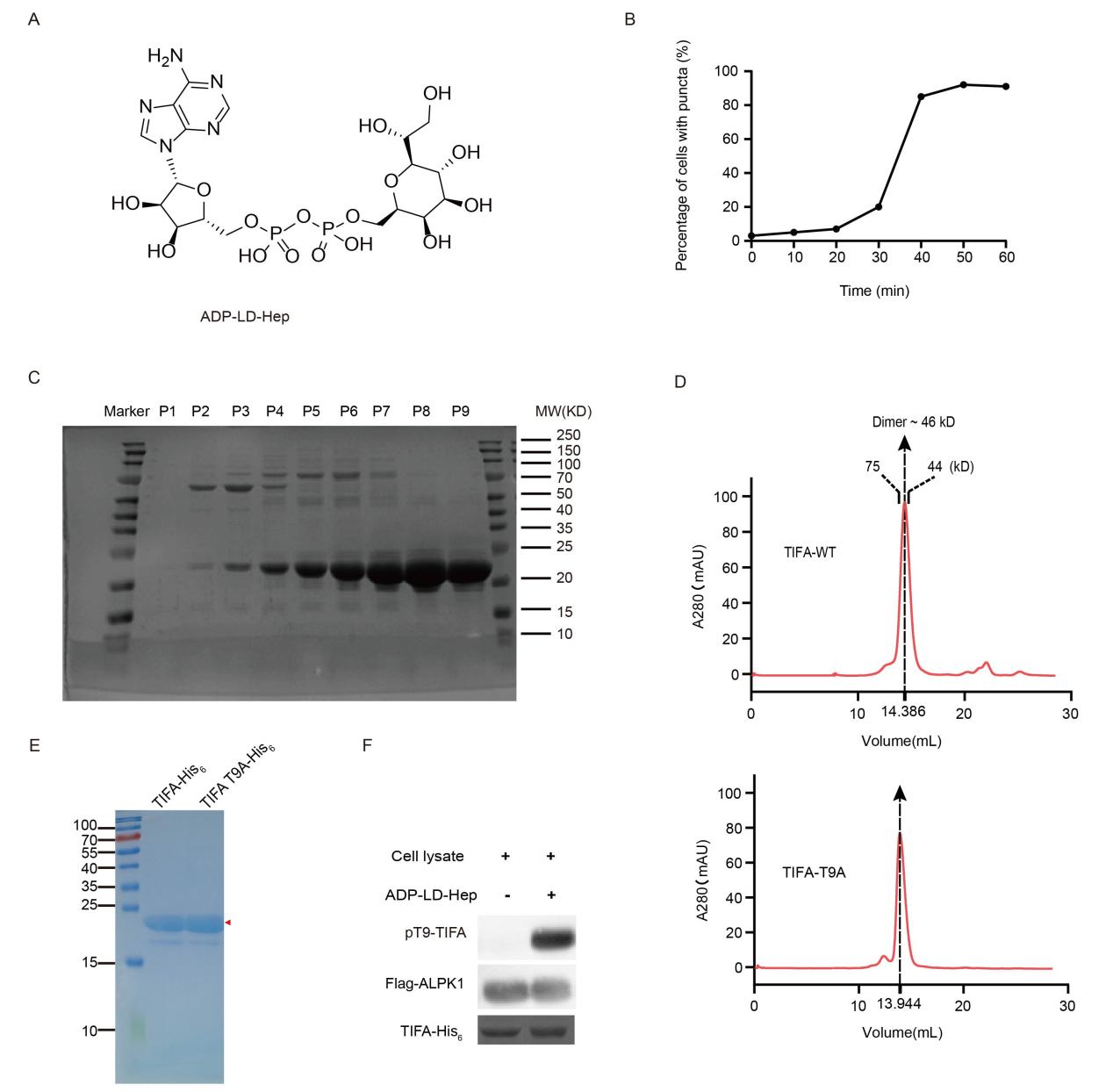


**Fig. S1.** TIFA forms liquid-like condensates stimulated with ADP-heptose. (A) Chemical structure of ADP-LD-Hep. (B) Percentage change of HEK 293T cells with GFP-TIFA puncta in a time course treatment of ADP-LD-Hep at 10 µM. ***, *P*< 0.001. (C) The affinity-purified TIFA was further purified by a Supedex 200 Increase 10/300 GL column and the fractions were analyzed by Coomassie blue staining. Fractions P8 and P9 were used to conduct the in vitro experiments (D) TIFA exists as a dimer. TIFA and TIFA T9A were separated by size-exclusion column Superdex 200 Increase 10/300 GL. (E) Coomassie blue staining of purified TIFA-His_6_ (23 kDa) and TIFA-T9A-His_6_ (23 kDa). (F) 293T cell lysates containing overexpressed Flag-ALPK1 (in cytoplasm) were incubated with TIFA-His_6_ in the presence of ADP-LD-Hep. Western blotting result showing the phosphorylation of TIFA upon addition of ADP-LD-Hep.


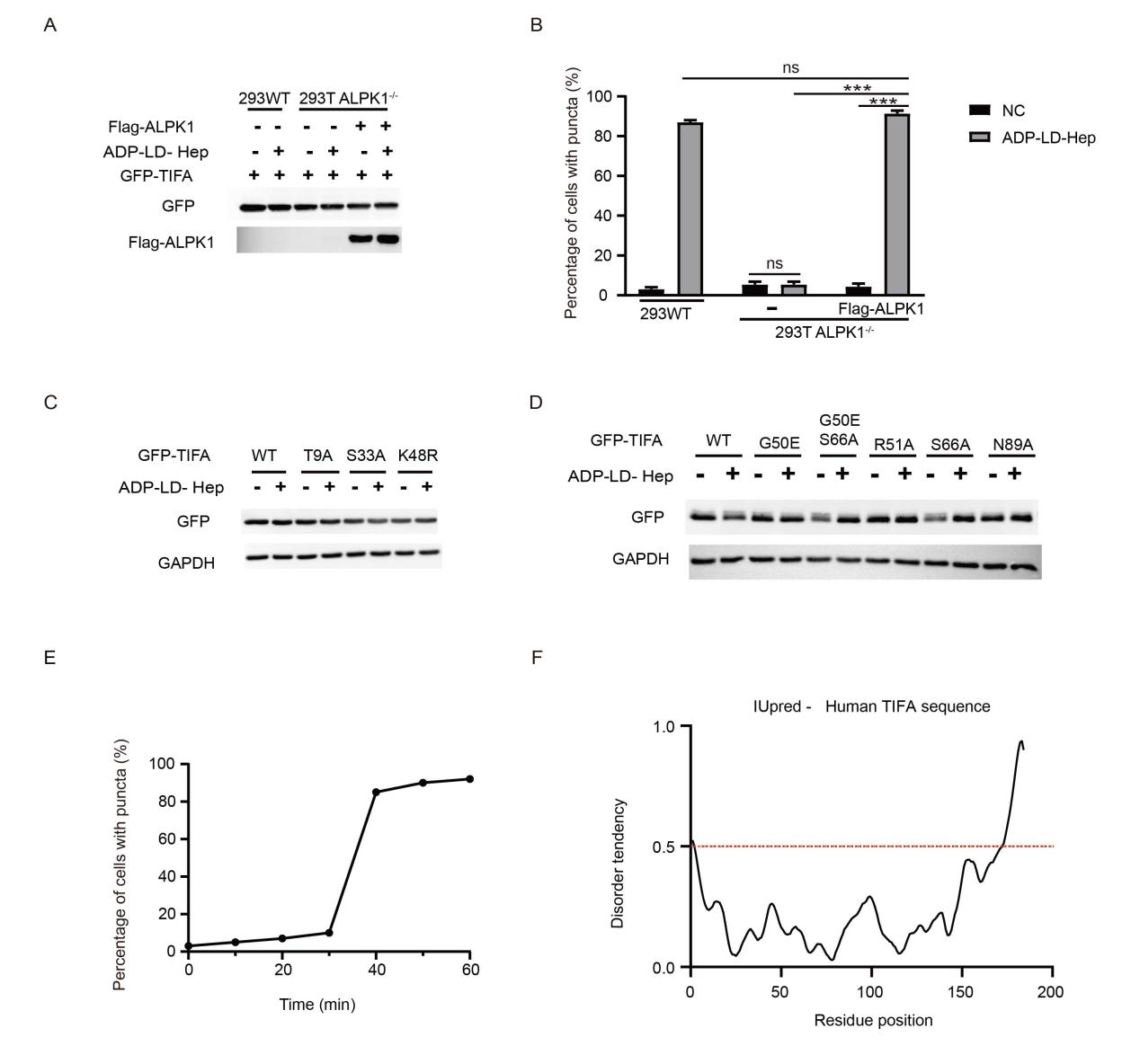


**Fig. S2.** ALPK1, the pT9-FHA domain, and the IDR segment are necessary for TIFA liquid condensation. (A) Protein expression level of GFP-TIFA and Flag-ALPK1 in 293T cells (Wild-type or *ALPK1*^−/−^ 293T cells). (B) Statistics of puncta in cells (Wild-type or *ALPK1*^−/−^ 293T cells) overexpressing GFP-TIFA with treatment of ADP-LD-Hep at 10 µM. ***, *P*< 0.001. (C and D) Protein expression level of GFP-TIFA mutant in 293T cells. (E) Percentage change of 293T cells with GFP-TIFA-E178A puncta in a time course treatment of ADP-LD-Hep at 10 µM. ***, *P*< 0.001. (F) The disorder tendency of human TIFA as predicted by IUpred (<https://iupred.elte.hu/>).


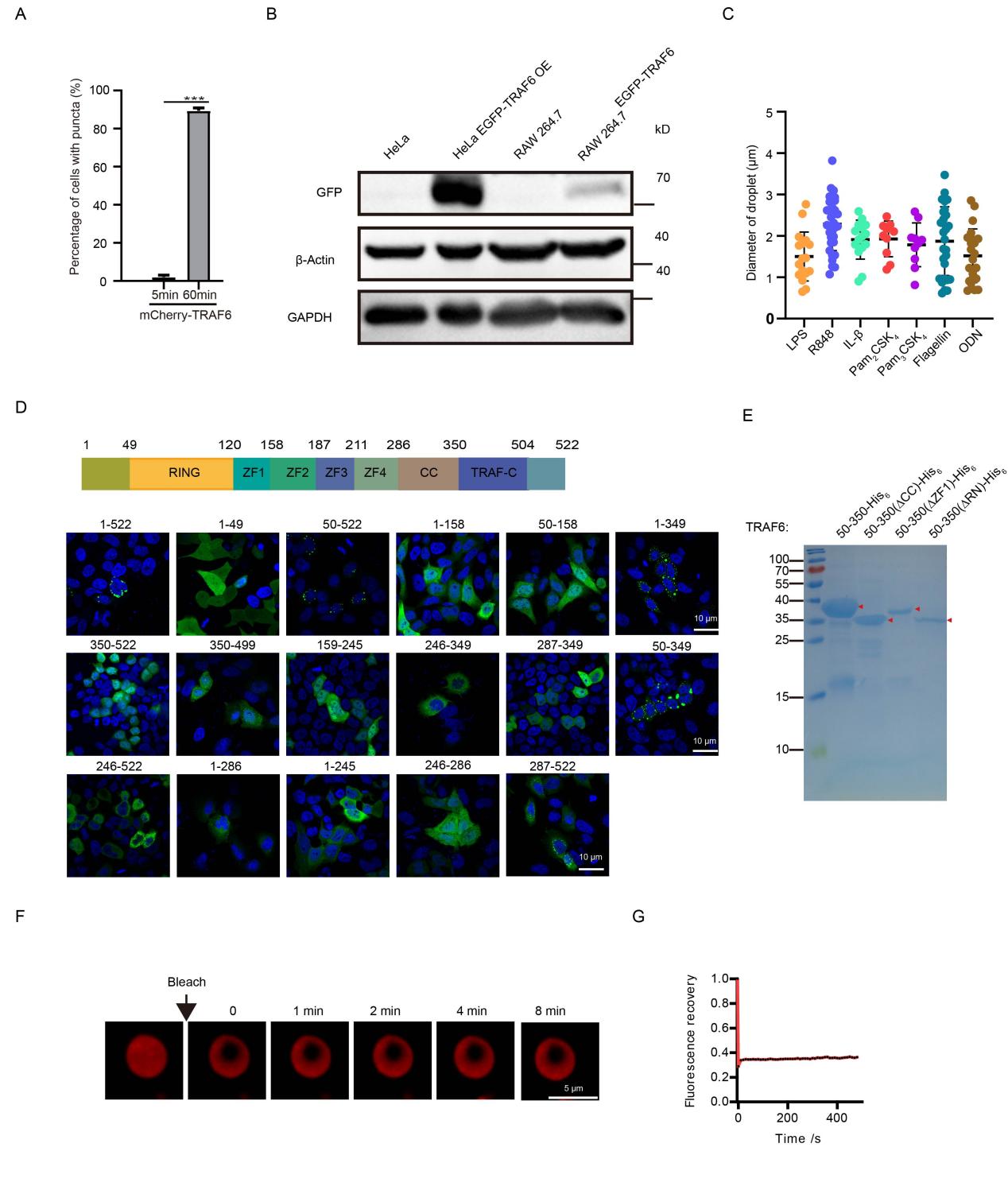


**Fig. S3.** The phenomenon of TRAF6 undergoing phase separation is universal. (A) Percentage changes of 293T-mCherry-TRAF6/ cells stimulated with ADP-LD-Hep at 10 µM. ***, *P*< 0.001. (B) Comparision of EGFP-TRAF6 overexpressed in HeLa cells and EGFP-TRAF6 in Raw^EGFP-TRAF6^ cell lines. (C) The diameter distribution of the droplets formed by LPS, R848, IL-1β，Pam_2_CSK_4_, Pam_3_CSK_4_, Flagellin, ODN stimulations. (D) Representative images of TRAF6 truncations in HeLa cells. (E) Coomassie blue staining of purified TRAF6(50-350)-His_6_ (38 kDa), TRAF6(50-350ΔCC)-His_6_ (30 kDa), TRAF6(50-350ΔZF1)-His_6_ (34 kDa) and TRAF6 (50-350ΔRN)-His_6_ (31 kDa). (F) Representative FRAP images of TRAF6 condensates. (G) Statistic analysis of FRAP in (F).

**
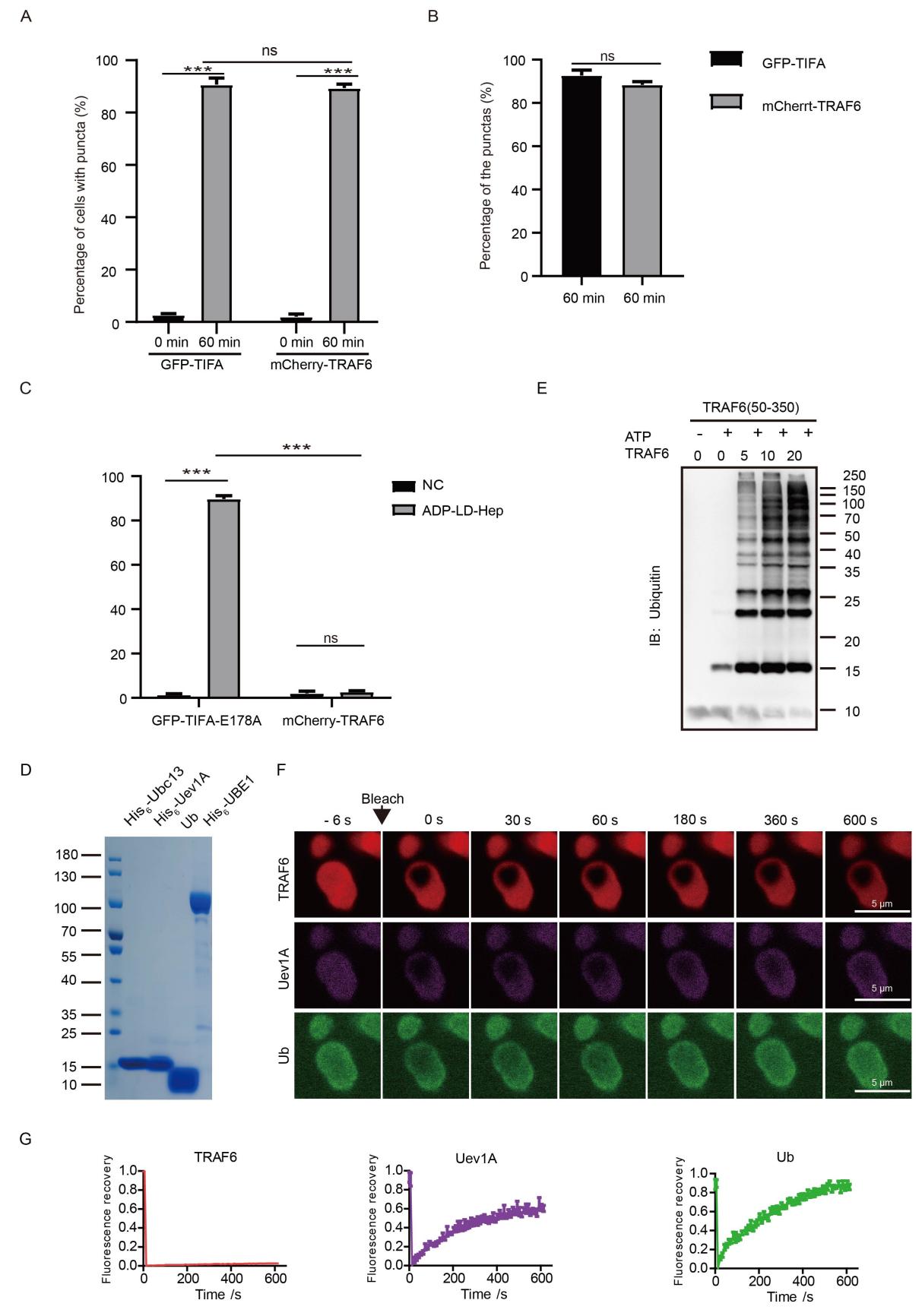
Fig. S4.** TRAF6 is recruited in the TIFA condensates and promotes K63 poly-ubiquitin chain synthesis. (A and B) Percentage changes of 293T-mCherry-TRAF6/GFP-TIFA cells stimulated with ADP-LD-Hep at 10 µM. ***, *P*< 0.001. (C) Percentage change of 293T-mCherry-TRAF6/GFP-TIFA-E178A cells stimulated with ADP-LD-Hep at 10 µM. ***, *P*< 0.001. (D) Coomassie blue staining of purified His_6_-UBE1 (118 kDa), His_6_-Ubc13 (17 kDa), Ub (9kDa) and His_6_-Uev1A (17 kDa). (E) Western blotting of TRAF6(50-350) ubiquitination reactions in vitro. (F) Representative images of fluorescence recovery of a TRAF6, Uev1A and Ub condensate. (G) FRAP analysis assaying the exchange kinetics of each protein between the TRAF6 condensates and the solution in E. The concentrations of TRAF6, Ubc13, Uev1A, Ub and E1 were 20 μM, 1 μM, 1 μM, 50 μM and 0.1 μM separately in (F-G). ATP concentration was 2 mM in (E-F).

**
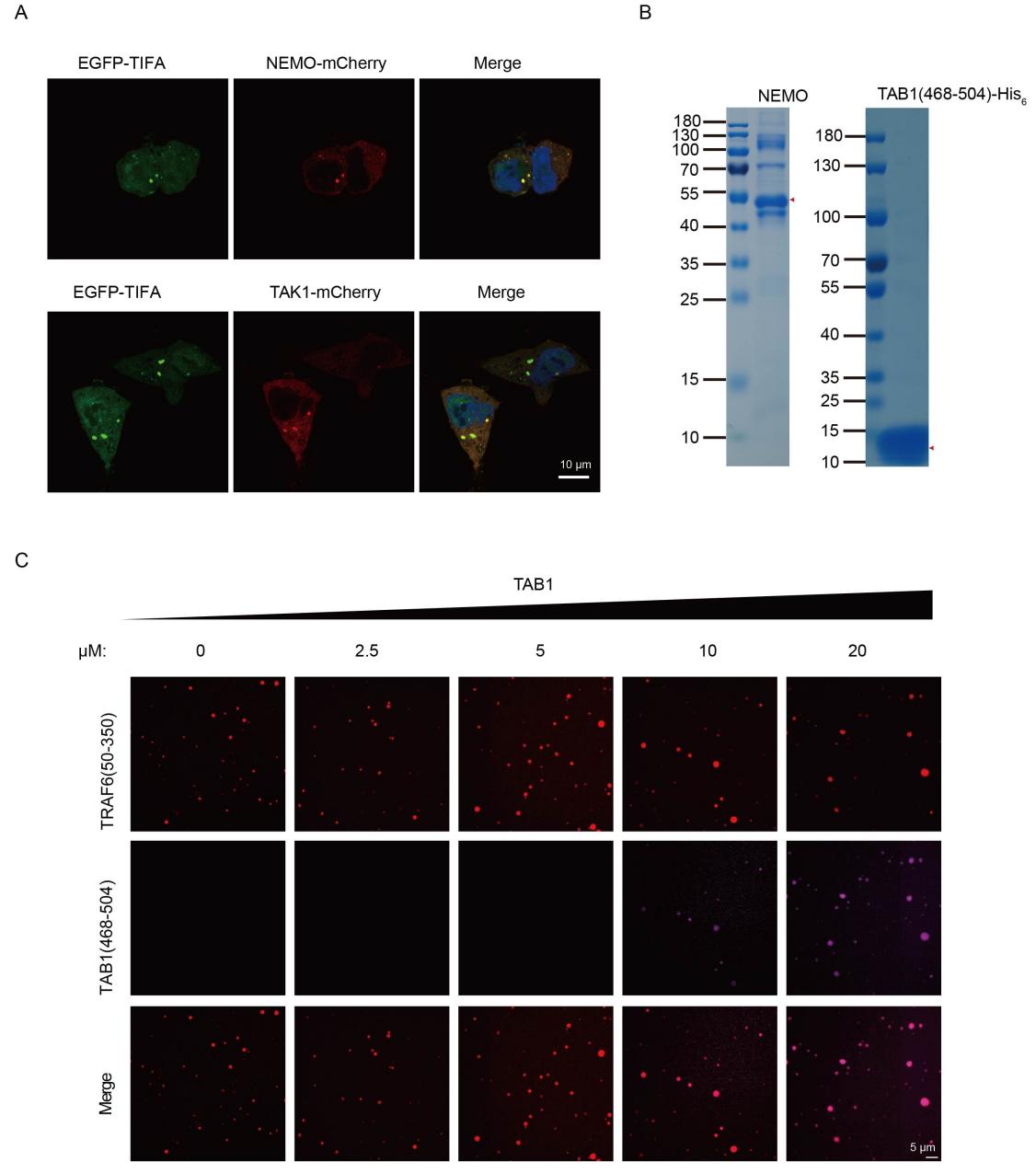
**

**Fig. S5**. TIFA and TRAF6 phase separation recruits and activates downstream proteins. (A) Representative images of EGFP-TIFA co-expressed with TAK1-mCherry and NEMO-mCherry separately in 293T cells. (B) Coomassie blue staining of purified NEMO (49 kDa) and TAB1(468-504)-His_6_ (8 kDa). (C) Representative colocalization images of TRAF6 and TAB1 in vitro at indicated concentrations. The concentration of TRAF6 in (C) is 5 μM.

**
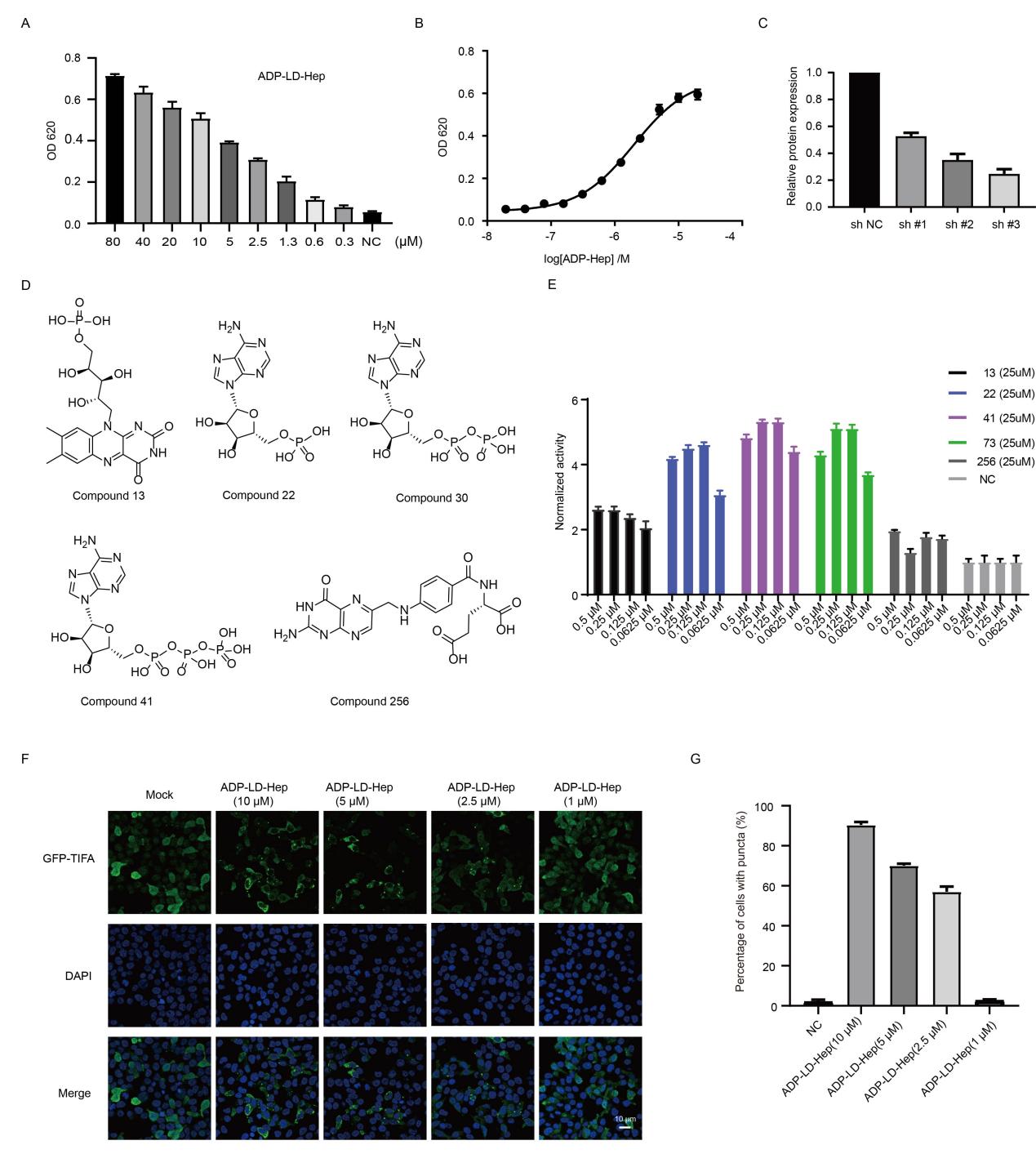
**

**Fig. S6.** TIFA LLPS promotes the activation of the ALPK1/TIFA signaling pathway. (A and B) ADP-Hep dose-dependently activates NF-κB activation with an IC_50_ of 2.3 µM. (C) Statistic analysis of TIFA protein knockdown in 293T cells. #No. 3 shRNAs showed about 70% knockdown of TIFA. The results were average of three independent replicates. (D) Chemical structures of screening compounds: **13, 22, 30, 41, 256**. (E) Normalized data on the ability of compounds (**13, 22, 30, 41, 256**) to activate NF-κB activation. (F) Represent images of GFP-TIFA puncta after ADP-LD-Hep stimulation at different doses for 2h. Scale bar, 10 µm. (G) Percentage change of 293T cells with GFP-TIFA puncta treatment with different doses of ADP-LD-Hep. ***, *P*< 0.001.

**Table S1. Materials.**

| **REAGENT or RESOURCE** | **SOURCE** | **IDENTIFIER** |
| --- | --- | --- |
| **Antibodies** | | |
| TIFA(phospho T9) | Abcam | ab214815 |
| p-IκBα | Sigma-Aldrich | ZRB1554 |
| Anti-Flag tag pAb | MBL | PM020 |
| GAPDH (14C10) | Cell Signaling Tech | 2118S |
| *β*-tubulin | Invitrogen | PA5-86259 |
| Rabbit monoclonal anti-GFP(D5.1) | Cell Signaling Technology | Cat#: 2956 |
| Mouse monoclonal anti-Ubiquitin(P4D1) | Santa Cruz Biotechnology | Cat#: sc-8017 |
| Rabbit monoclonal anti-Ubiquitin, Lys63-specific(Apu3) | Sigma-Aldrich | Cat#: 05-1308 |
| Mouse monoclonal anti-IKKγ(NEMO) | BD Biosciences | Cat#: 611306 |
| Rabbit polyclonal anti-p-TAK1(T187) | Cell Signaling Technology | Cat#: 4536 |
| Rabbit recombinant anti-TRAF6(D21G3) | Cell Signaling Technology | Cat#: 8028 |
| Goat anti-Mouse IgG (H+L) Highly Cross-Adsorbed Secondary Antibody, Alexa Fluor™ 568 | Invitrogen | Cat#: A-11031 |
| Goat anti-Rabbit IgG (H+L) Cross-Adsorbed Secondary Antibody, Alexa Fluor™ 568 | Invitrogen | Cat#: A-11011 |
| Fast Mutagenesis System | TransGenBiotech | Cat#: FM111-01 |
| System | | |
| TransSafe™ Mycoplasma Prevention Reagent | TransGen Biotech | Cat#:FM501-01 |
| **Bacterial and virus strains** | | |
| E. coli BL21 (DE3) | TransGenBiotech | CD601-02 |
| E. coli *Transetta* (DE3) | TransGenBiotech | CD801-02 |
| E. coli Trans5α | TransGenBiotech | CD201-01 |
|  |  |  |
| **Chemicals, peptides, and recombinant proteins** | | |
| ADP-Heptone | J&K scientific | 9020852 |
| Compound 22 | Center of Pharmaceutical Technology, Tsinghua University |  |
| Protease Inhibitor Cocktail | Thermo Fisher Scientific | Cat#78430 |
| Phosphatase inhibitor | Thermo Fisher Scientific | Cat#A32957 |
| Anti-Flag affinity gel | Bimake | Cat#B23102 |
| TRIzol Reagent | Invitrogen | Cat#15596018 |
| Streptavidin Sepharose-R Beads | Cell Signaling Tech | Cat#3419S |
| Pierce RIPA Buffer | Thermo Fisher Scientific | Cat#89900 |
| DAPI | Beyotime | Cat#C1006 |
| Cyanine 3 monosuccinimidyl ester | AAT Bioquest | Cat#: AAT-141 |
| Cyanine 5 monosuccinimidyl ester | AAT Bioquest | Cat#: AAT-151 |
| iFluor® 488 maleimide | AAT Bioquest | Cat#: AAT-1062 |
| LPS | Sigma-Aldrich | Cat#: L2630 |
| R848 | InvivoGen | Cat#: tlrl-r848 |
| Pam_2_CSK_4_ | InvivoGen | Cat#: tlrl-pm2s-1 |
| Pam_3_CSK_4_ | InvivoGen | Cat#: tlrl-pms |
| Flagellin | InvivoGen | Cat#:tlrl-bsfla |
| ODN 1668 | InvivoGen | Cat#: tlrl-1668 |
| IL-1β | novoprotein | Cat#:C042 |
| C25-140 | TargetMol | Cat#:T7889 |
| **Critical commercial assays** | | |
| Lipofectamine 3000 Transfection Kit | Invitrogen | Cat#2145954 |
| HOOK(TM) Dye Labeling Kit (5/6) TAMRA-SE (Rhodamine) | Sangon biotech | Cat#C006142-0005 |
| HOOK(TM) Dye Labeling Kit (FITC) | Sangon biotech | Cat#C006141-0005 |
| iScript cDNA synthesis kit | Bio-Rad | Cat#1706691 |
| iTaq Universal SYBR Green Supermix | Bio-Rad | Cat#172521 |
| Pierce BCA Protein Assay Kit | Thermo Fisher Scientific | Cat#23225 |
| Fast Mutagenesis System | Transgen | Cat#FM111 |
| pEASY-Uni Seamless Cloning and Assembly Kit | Transgen | Cat#CU101 |
| SuperSignal™ West Pico PLUS Substrate Kit | Thermo Fisher Scientific | Cat#34578 |
| **Experimental models: Cell lines** | | |
| HEK293T | ATCC | Cat#CRL-3216 |
| HEK293T/17 | ATCC | Cat#: CRL-11268 |
| HeLa | ATCC | Cat#: CCL-2 |
| RAW 264.7 | ATCC | Cat#:TIB-71 |
| **Recombinant DNA** | | |

| pCMV-3Tag (Flag) | Agilent Technologies | #240195 |
| --- | --- | --- |
| lentiCRISPR-V2 | Addgene | #52961 |
| pMD2.G | Addgene | #12259 |
| psPAX2 | Addgene | #12260 |
| pET22b- 6×His tag | Dr. Feng Shao | N/A |
| pTY-EGFP-TIFA | This manuscript | N/A |
| pCMV-Flag-TIFA (T9A) | This manuscript | N/A |
| pCMV-Flag- TIFA (S33A) | This manuscript | N/A |
| pCMV-Flag- TIFA (K48R) | This manuscript | N/A |
| pCMV-Flag- TIFA (G50E) | This manuscript | N/A |
| pCMV-Flag- TIFA (R51A) | This manuscript | N/A |
| pCMV-Flag- TIFA (S66A) | This manuscript | N/A |
| pCMV-Flag- TIFA (N89A) | This manuscript | N/A |
| pTY-mCherry-ALPK1 | This manuscript | N/A |
| pTY-EGFP-TIFA (T9A) | This manuscript | N/A |
| pTY-EGFP-TIFA (S33A) | This manuscript | N/A |
| pTY-EGFP-TIFA (K48R) | This manuscript | N/A |
| pTY-EGFP-TIFA (G50E) | This manuscript | N/A |
| pTY-EGFP-TIFA (R51A) | This manuscript | N/A |
| pTY-EGFP-TIFA (S66A) | This manuscript | N/A |
| pTY-EGFP-TIFA (N89A) | This manuscript | N/A |
| pTY-EGFP-TIFA (1-150) | This manuscript | N/A |
| pTY-EGFP-TIFA (1-160) | This manuscript | N/A |
| pTY-EGFP-TIFA (1-170) | This manuscript | N/A |
| pTY-EGFP-TIFA (ΔFA-C) | This manuscript | N/A |
| pTY-EGFP-TIFA (ΔN+C) | This manuscript | N/A |
| pTY-EGFP-TIFA (ΔN) | This manuscript | N/A |
| pTY-EGFP-TIFA (ΔFA) | This manuscript | N/A |
| pMD2.G | Addgene | #12259 |
| EGFP-TRAF6 | This manuscript | N/A |
| EGFP-TRAF6 50-349 | This manuscript | N/A |
| EGFP-TRAF6^ΔRN^ | This manuscript | N/A |
| EGFP-TRAF6^ΔZF1^ | This manuscript | N/A |
| EGFP-TRAF6^ΔZF2^ | This manuscript | N/A |
| EGFP-TRAF6^ΔZF3^ | This manuscript | N/A |
| EGFP-TRAF6^ΔZF4^ | This manuscript | N/A |
| EGFP-TRAF6^ΔCC^ | This manuscript | N/A |
| EGFP-TRAF6(121-349) | This manuscript | N/A |
| EGFP-TRAF6(50-349)^ΔZF1^ | This manuscript | N/A |
| EGFP-TRAF6(50-349)^ΔZF2^ | This manuscript | N/A |
| EGFP-TRAF6(50-349)^ΔZF3^ | This manuscript | N/A |
| EGFP-TRAF6(50-349)^ΔZF4^ | This manuscript | N/A |
| EGFP-TRAF6(50-286) | This manuscript | N/A |
| TAK1-mCherry | This manuscript | N/A |
| TAB1-mCherry | This manuscript | N/A |
| TAB3-mCherry | This manuscript | N/A |
| NEMO-mCherry | This manuscript | N/A |
| IKKα-mCherry | This manuscript | N/A |
| IKKβ-mCherry | This manuscript | N/A |
| GST-NEMO | This manuscript | N/A |
| TRAF6(50-350)-His_6_ | This manuscript | N/A |
| TRAF6(121-350)-His_6_ | This manuscript | N/A |
| TRAF6(50-286)-His_6_ | This manuscript | N/A |
| TAB1(468-504)-His_6_ | This manuscript | N/A |
| His_6_-Uev1A/His_6_-UBE2V1 | This manuscript | N/A |
| His_6_-MBP-SUMO-Ub | Li Yu Lab |  |
| His_6_-Ubc13/UBE2N | Li Yu Lab |  |
| His_6_-UBE1 | Lei Liu Lab |  |
| **Software and algorithms** | | |
| GraphPad Prism 7 | https://www.graphpad.com/ | N/A |
| Adobe Illustrator CC | https://www.adobe.com/products/illustrator.html | N/A |
| NIS-Elements Viewer 5.21 | https://www.microscope.healthcare.nikon.com | N/A |
| ImageJ 1.53c | https://imagej.nih.gov | N/A |
| Jalview 2.11.1.7 | http://www.jalview.org/ | N/A |
| Imaris 9.6.0 | Imaris.oxinst.com | N/A |

**Table S2. Primers used for quantitative PCR.**

| qPCR Primers | Sequences (5’ to 3’) |
| --- | --- |
| *GAPDH-F* | ATGACATCAAGAAGGTGGTG |
| *GAPDH-R* | CATACCAGGAAATGAGCTTG |
| *ALPK1-F* | ATGAATAATCAAAAAGTGGTAG |
| *ALPK1-R* | CTATGTGCATGGTTTCTCCA |
| *IL-8-F* | AATCTGGCAACCCTAGTCTGCTA |
| *IL-8-R* | AAACCAAGGCACAGTGGAACA |
