# Supplementary figures and images for: ADP-Hep-Induced Liquid Phase Condensation of TIFA-TRAF6 Activates ALPK1/TIFA-Dependent Innate Immune Responses
Source: Research (Wash D C). 2024 Feb 14;7:0315. doi: 10.34133/research.0315 (PMC10865109; doi:10.34133/research.0315)

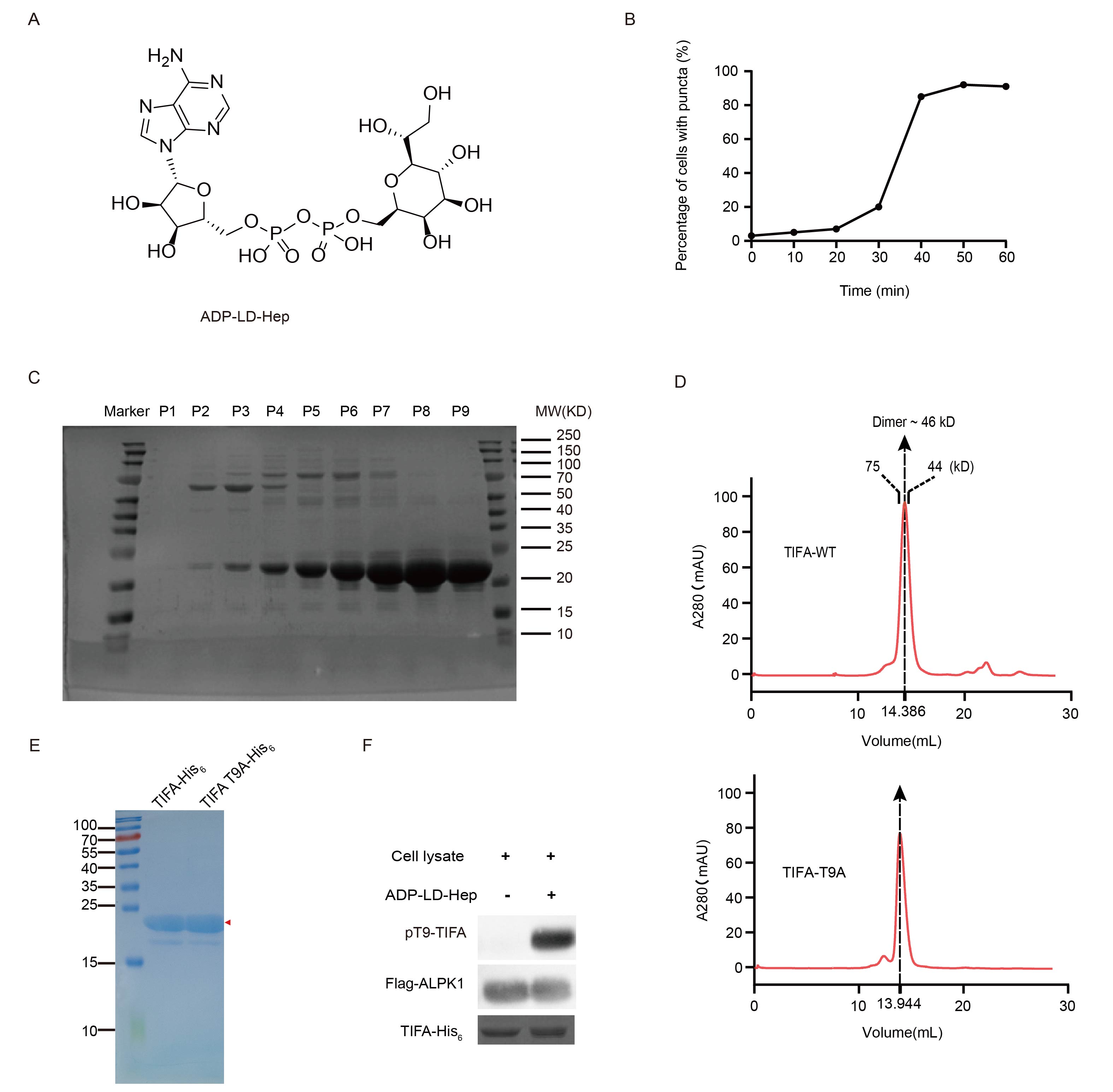

Supplement: Supplementary 1 — Figs. S1 to S6 Tables S1 and S2 [file research.0315.f1.zip › Figure S1.jpg]

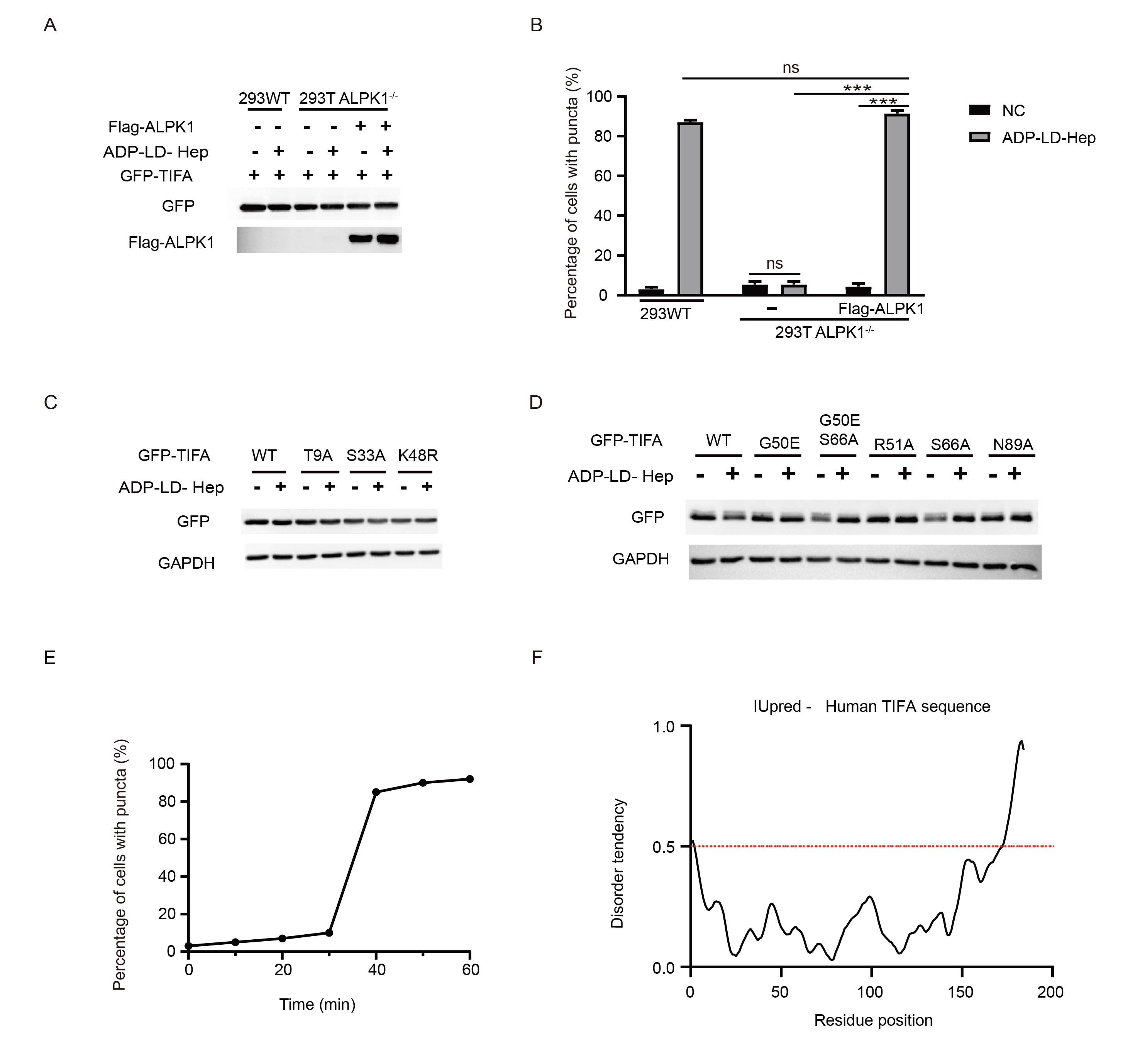

Supplement: Supplementary 1 — Figs. S1 to S6 Tables S1 and S2 [file research.0315.f1.zip › Figure S2.jpg]

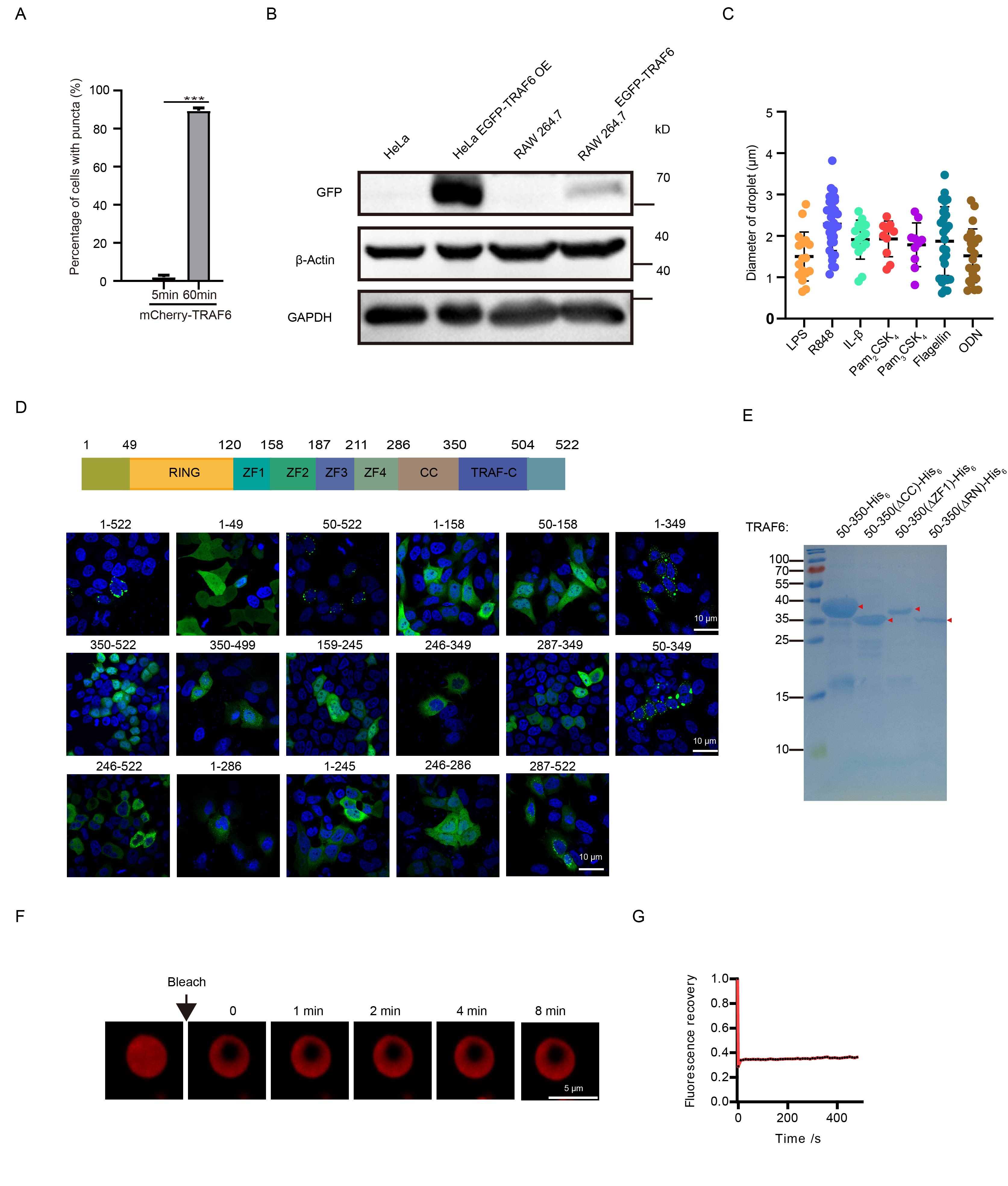

Supplement: Supplementary 1 — Figs. S1 to S6 Tables S1 and S2 [file research.0315.f1.zip › Figure S3.jpg]

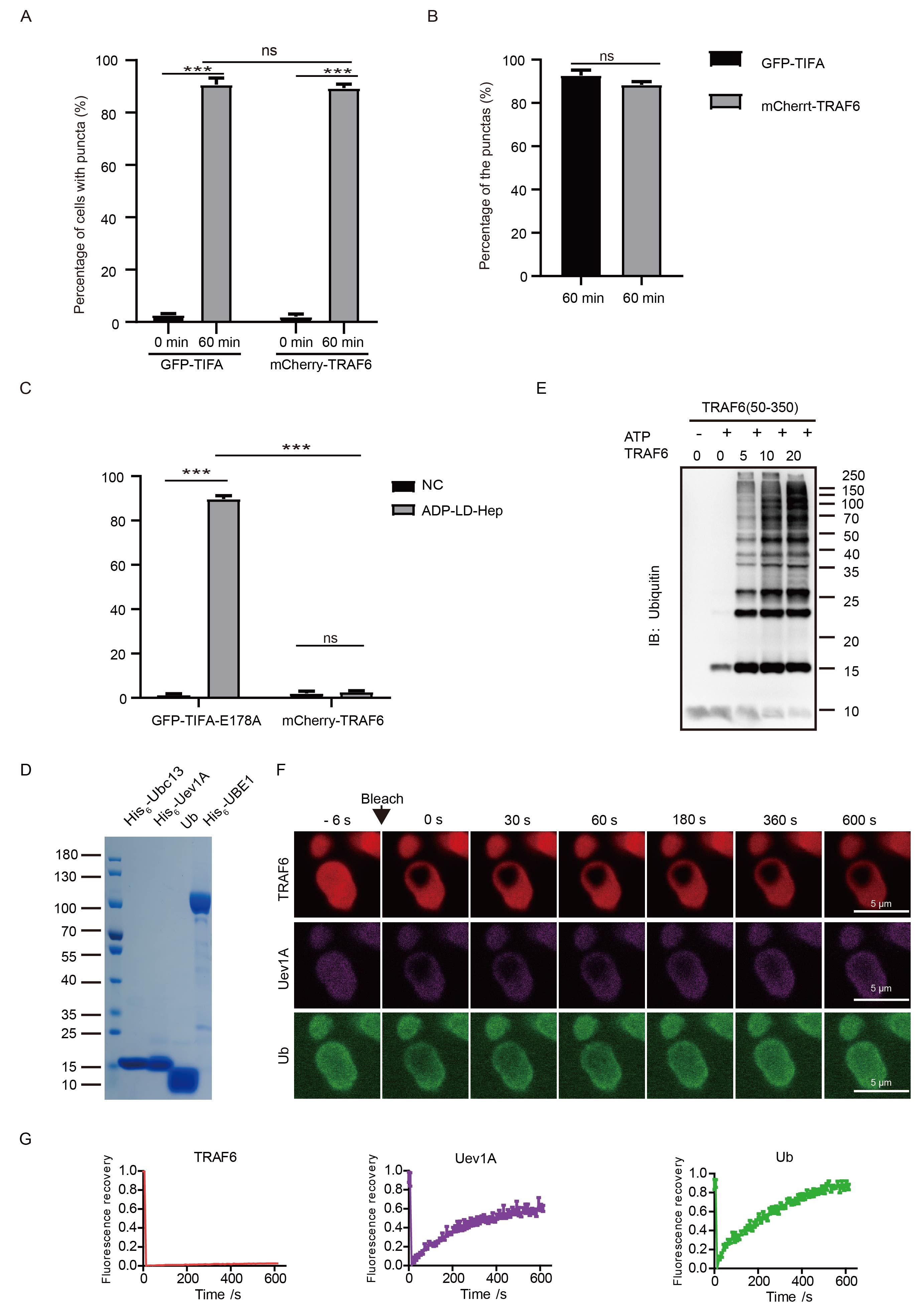

Supplement: Supplementary 1 — Figs. S1 to S6 Tables S1 and S2 [file research.0315.f1.zip › Figure S4.jpg]

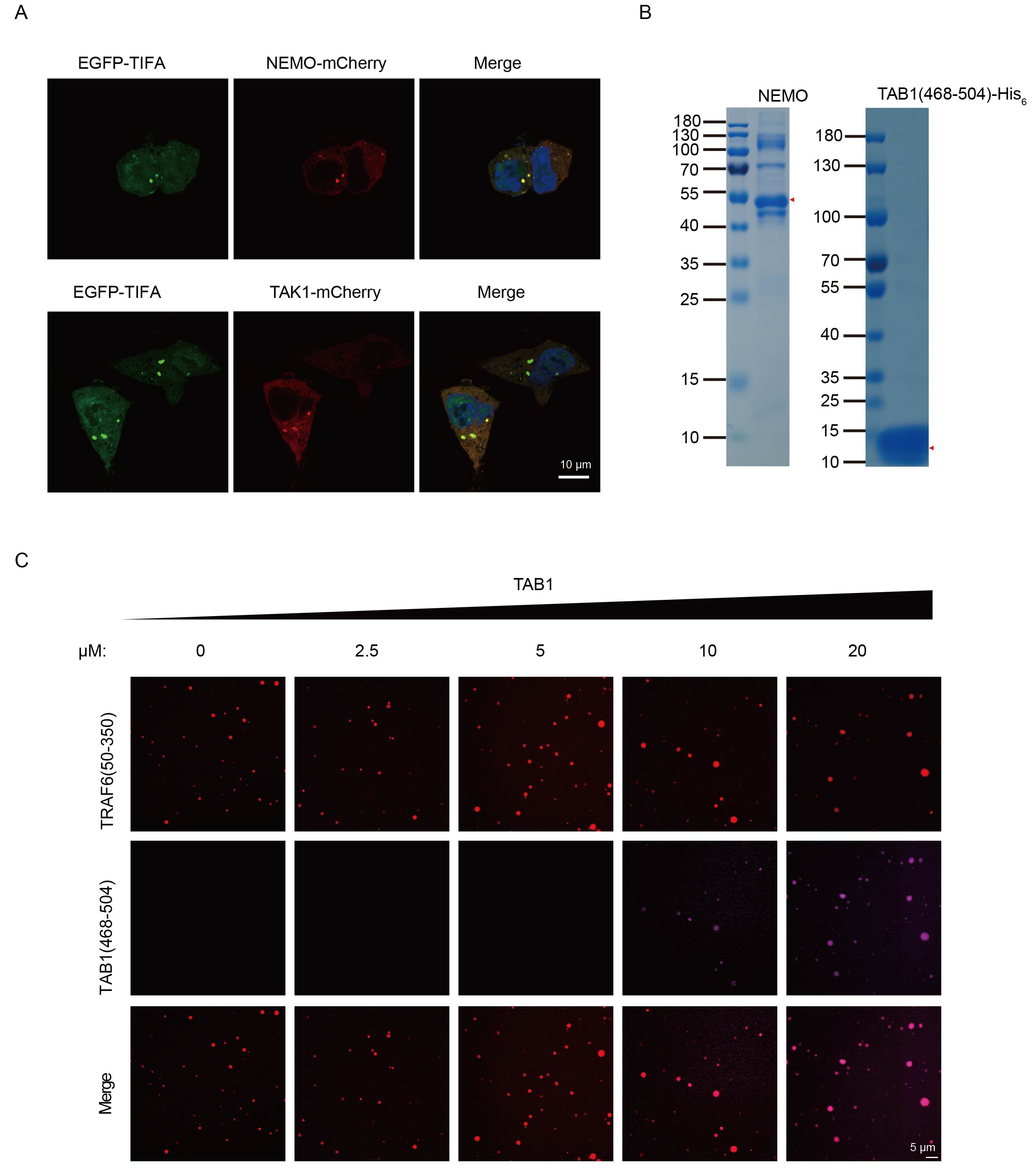

Supplement: Supplementary 1 — Figs. S1 to S6 Tables S1 and S2 [file research.0315.f1.zip › Figure S5.jpg]

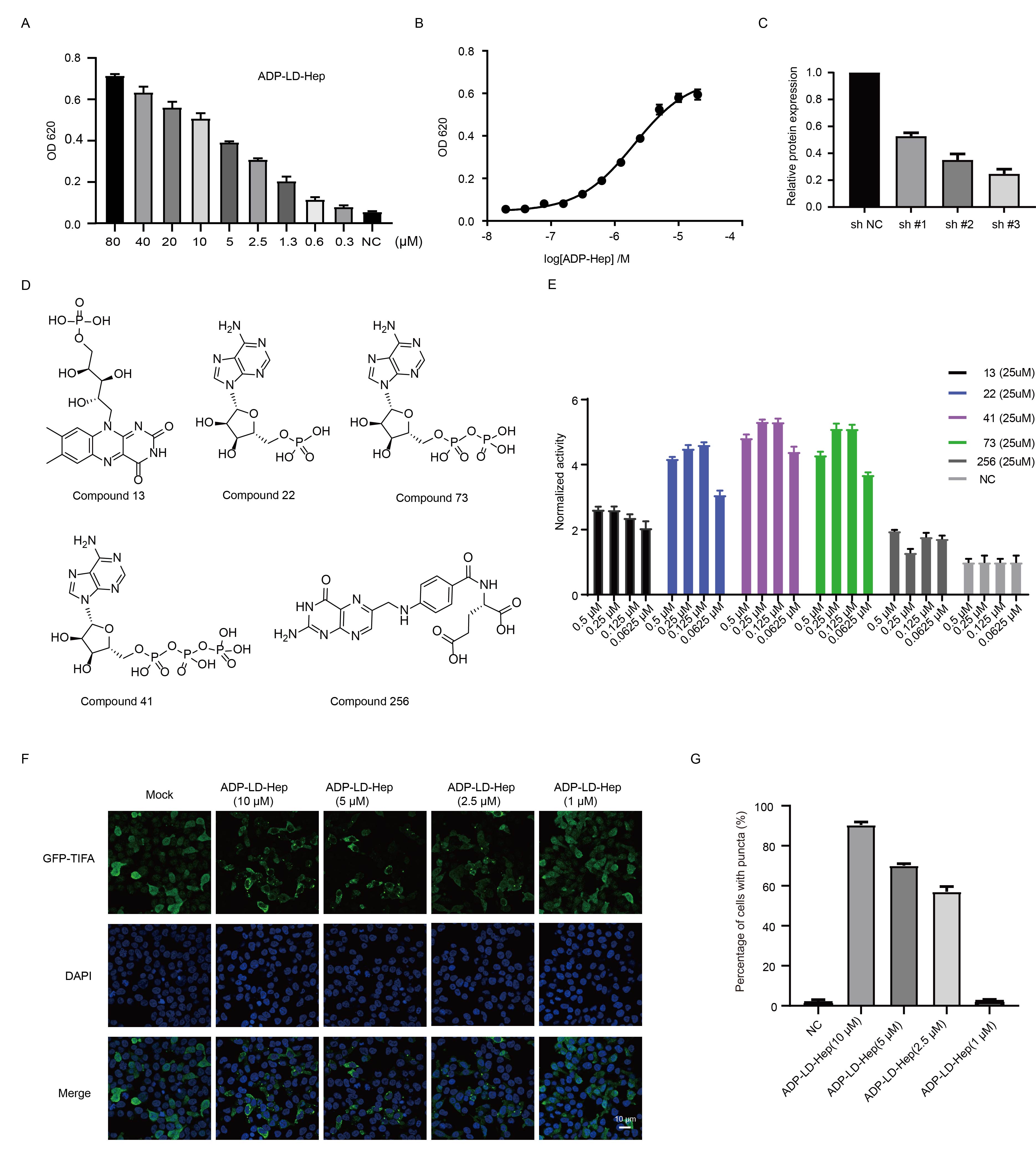

Supplement: Supplementary 1 — Figs. S1 to S6 Tables S1 and S2 [file research.0315.f1.zip › Figure S6.jpg]
